# Comparative efficacy of Chinese herbal injections for treating acute cerebral infarction: a network meta-analysis of randomized controlled trials

**DOI:** 10.1186/s12906-018-2178-9

**Published:** 2018-04-03

**Authors:** Shi Liu, Jia-Rui Wu, Dan Zhang, Kai-Huan Wang, Bing Zhang, Xiao-Meng Zhang, Di Tan, Xiao-Jiao Duan, Ying-Ying Cui, Xin-Kui Liu

**Affiliations:** Department of Clinical Chinese Pharmacy, School of Chinese Materia Medica, Beijing University of Chinese Medicine, Beijing, 100102 China

**Keywords:** Network meta-analysis, Acute cerebral infarction, Chinese herbal injection

## Abstract

**Background:**

Chinese herbal injections (CHIs) are prepared by extracting and purifying effective substances from herbs (or decoction pieces) using modern scientific techniques and methods. CHIs combined with aspirin + anticoagulants + dehydrant + neuroprotectant (AADN) are believed to be effective for the treatment of acute cerebral infarction (ACI). However, no randomized controlled trial (RCT) has been performed to directly compare the efficacies of different regimens of CHIs. Therefore, we performed a systematic review and network meta-analysis (NMA) to compare the efficacies of different regimens of CHIs for ACI.

**Methods:**

We conducted an overall and systematic retrieval from literature databases of RCTs focused on the use of CHIs to treat ACI up to June 2016. We used the Cochrane Handbook version 5.1.0 and CONSORT statement to assess the risk of bias. The data were analyzed using STATA 13.0 and WinBUGS 1.4.3 software.

**Results:**

Overall, 64 studies with 6225 participants involving 15 CHIs were included in the NMA. In terms of the markedly effective rate, Danhong (DH) + AADN had the highest likelihood of being the best treatment. In terms of the improvement of neurological impairment, Shuxuening (SXN) + AADN had the highest likelihood of being the best treatment. Considering two outcomes, injections of SXN, Yinxingdamo (YXDM), DH, Shuxuetong (SXT), HongHuaHuangSeSu (HHHSS), DengZhanXiXin (DZXX) and Shenxiong glucose (SX) plus AADN were the optimum treatment regimens for ACI, especially SXN + AADN and YXDM + AADN.

**Conclusions:**

Based on the NMA, SXN, YXDM, DH, SXT, HHHSS, DZXX and SX plus AADN showed the highest probability of being the best treatment regimens. Due to the limitations of the present study, our findings should be verified by well-designed RCTs.

**Electronic supplementary material:**

The online version of this article (10.1186/s12906-018-2178-9) contains supplementary material, which is available to authorized users.

## Background

Acute cerebral infarction (ACI) is one of the most common cerebral vascular diseases, also referred to as ischemic stroke, which is result from ischemia, hypoxia and cerebral blood circulation [1–3]. ACI has the characteristics of high disability and recurrence [4–6]. Besides, it is a major disease leading to serious damage of central nervous system or death [4, 7]. It was estimated that ACI cause 6.2 million mortalities annually worldwide [8]. In traditional Chinese medicine (TCM) theories, ACI refers to “apoplexy”, majorly due to blood stasis syndrome [3]. Therefore, promoting blood flow is of primary importance. It has been proven that TCM is an effective complementary intervention for stroke, especially in the treatment of ischemic stroke [9–13].

Currently, therapies for ACI include thrombolytics, antithrombotics, anticoagulants, and neuroprotectants [14]; this was the Grade-I recommendation in the guidance of diagnosis and treatment of acute ischemic stroke in China 2010, which fully considered the national conditions and clinical experience. Among them, thrombolytic has a short therapeutic time window. Thus, many patients easily miss the effective time window of thrombolysis. ACI patients who are not eligible for thrombolysis therapy should be given oral aspirin, which was approved by the US Food and Drug Administration (FDA), at a dose ranging from 150 to 300 mg/d as soon as possible (level of evidence: A) [15, 16]. And patients with brain edema could use a mannitol intravenous drip. Although more high-quality clinical trials are needed to further demonstrate the efficacy and safety of neuroprotective agents, a number of RCTs have suggested that edaravone and cerebroprotein hydrolysate improve functional outcomes and the safety of patients with ACI [17–20]. Additionally, the therapeutic principle of invigorating blood circulation for removing blood stasis of TCM holds a significant position for ACI. Chinese herbal injections (CHIs) have the characteristics of rapid efficacy and high bioavailability [21–23].Presently, 37 injections are often used in the treatment of cerebral infarction, such as Xueshuantong injection, Shuxuening injection, Mailuoning injection, and Danshenchuanxiongqin injection [24–27]. Clinical data [28–32] from systematic reviews of RCTs have demonstrated the beneficial effects of CHIs for inhibiting platelet aggregation, improving blood microcirculation and nerve function, enhancing the tolerance of ischemic tissue to hypoxia, and protecting against ischemic reperfusion injury.

Hence, this study systematically evaluated the clinical effectiveness of CHIs combined with an aspirin + anticoagulants + dehydrant + neuroprotectant (AADN) regimen in ACI patients that conformed to the standardized treatment of ischemic cerebrovascular disease: integration, individualization and sequencing. However, there is no direct head-to-head evidence revealing the best CHIs for ACI treatment. Determining the superiority of a treatment based on a pairwise comparison meta-analysis is difficult. A network meta-analysis (NMA), which is an extension of a traditional meta-analysis, synthesizes the available evidence to enable simultaneous comparisons of different treatment options that lack direct head-to-head evaluations [33–35]. Therefore, the present study performed a NMA to compare the clinical efficacy of 37 CHIs combined with the AADN regimen to reveal the best CHIs for ACI, aiming to provide more sights for selection of ACI.

## Methods

### Eligibility criteria

Studies meeting the following criteria were included: (1) Clinical randomized controlled trials (RCTs) using CHIs + AADN to treat ACI regardless of blinding. (2) Cerebrovascular disease was diagnosed according to the standards revised by the Fourth National Conference on Cerebrovascular Disease by the Chinese Medical Association in 1995 [36]. The acute phase of ACI generally refers to 2 weeks after the onset of disease. Thus, this NMA enrolled patients with the course of disease within 2 weeks. No cerebral hemorrhage was detected using cranial computed tomography (CT) or magnetic resonance imaging (MRI). There were no limits on age, gender, race or disease severity. (3) Eligible comparisons were CHIs + AADN regimens versus the AADN regimen alone and CHIs + AADN regimens versus other CHIs + AADN regimens. There was no limitation on the dosages or treatment courses. (4) Outcome measures included the markedly effective rate, improvement of neurological impairment, activities of daily living function, and death from all causes within the treatment and during the entire follow-up period. The following formula was used: the markedly effective rate (%) = (number of recovered patients + number of patients with significant progress) / total number × 100%. The efficacy criteria were predominantly based on the reduction of the neurological deficit score and could be divided into four grades: recovery, significant progress, progress, and no change or deterioration. Recovery, significant progress, progress, and no change or deterioration were determined when the neurological deficit score decreased from 91% to 100%, between 46% and 90%, between 18% and 45%, and < 17%, respectively. The improvement of neurological impairment is expressed as the mean ± standard deviation.

The following studies were excluded: (1) studies that did not refer to the acute phase; (2) studies that did not meet the curative effect valuation standard; (3) studies involving patients who had a severe cognitive disorder, hemorrhagic tendency, or serious complications, such as atrial fibrillation, severe heart failure, severe liver and kidney diseases, undergoing surgery, acupuncture or other physical therapy; (4) data that were incorrect, incomplete or unavailable; (5) reviews or meta-analyses, experimental research, retrospective studies, case reports, and conference abstracts.

### Data sources and search strategy

A systematic literature search was performed using the following databases from inception to June 2016: PubMed, Cochrane Library, Embase, China National Knowledge Infrastructure Database (CNKI), Wanfang Database, and Chinese Biomedical Literature Database (CBM). The medical subject headings (MeSH) and free text words were used. No language or other restrictions were imposed. Furthermore, we hand searched the reference lists of all retrieved studies. The specific Chinese and English search terms for each CHIs are shown in Additional file 1: Table S1 and the detailed search strategy is shown in Additional file 2.

### Literature selection and data extraction

Two reviewers independently read the titles and abstracts of the literature to exclude literature that was obviously not relevant as well as reviews and pharmacological experiments. We retrieved the full text of the articles to determine whether they were eligible.

The data of interest from each included RCT were collected using a standard data abstraction form created in Microsoft Excel 2013 (Microsoft Corp, Redmond, WA, USA). The main components of the extracted data were as follows: (1) General information: author names and publication data; (2) Patient information: median age, number of patients, gender, and acute phase; (3) Intervention: names, dosages, and treatment; (4) Outcomes: the markedly effective rate, improvement of neurological deficit score, adverse drug reactions/adverse drug events (ADRs/ADEs), activities of daily living function, and death from all causes within the treatment and during the entire follow-up period.

### Quality assessment

The methodological quality of each included study was evaluated using the Cochrane Risk of Bias tool [37] and the CONSORT statement [38]. The items included randomization, blinding, dropout, eligibility criteria for participants, adverse events, and statistical methods. The judgments for each entry involved were divided into 3 grades: “high”, “unclear”, and “low”. A quality assessment was performed by two independent reviews, and disagreements were resolved by consensus.

### Statistical analysis

We performed a pairwise meta-analysis using STATA 13.0 software (Stata Corporation, College Station, TX, USA). The pooled odds ratios (*ORs*) were calculated for dichotomous data and standardized mean differences (*SMDs*) were calculated for continuous variables, both with 95% confidence interval (*95% CI*). The Chi-squared test was used to evaluate the heterogeneity between studies, and *I*^*2*^ was used to show the extent of heterogeneity. When *P* was ≥0.1 and *I*^*2*^ was ≤50%, no statistical heterogeneity was suggested and the Mantel-Haenszel fixed-effects model was used for the meta-analysis. When *P* < 0.1 and *I*^*2*^ was > 50%, we explored the sources of heterogeneity using a subgroup analysis and meta-regression. When there was no clinical heterogeneity, the Mantel-Haenszel random-effects model was used to perform the meta-analysis [37].

A Bayesian NMA was conducted using WinBUGS 1.4.3 software (MRC Biostatistics Unit, Cambridge, UK). The random-effects model with vague priors for multi-arm trials was used [39]. The model parameters were estimated using a Markov chain Monte Carlo method called Gibbs sampling. Convergence was found to be adequate after running 1000 samples. These samples were discarded as “burn-in,” and posterior summaries were based on 100,000 subsequent simulations. The results are reported as the *OR* and *SMD* with *95% CI*. To evaluate the inconsistency between direct and indirect effect estimates for the same comparison, we evaluated each closed loop in the network. In a closed loop, we employed the inconsistency factor (IF) to evaluate heterogeneity among the included studies. If the 95% CIs of the IF values were truncated at zero, it indicated that the 2 sources were in agreement [39]. To rank the treatments, we used the surface under the cumulative ranking probabilities (SUCRA); a SUCRA value of 100% is assigned to the best treatment and 0% for the worst treatment [39]. A comparison-adjusted funnel plot was used to assess the presence of small-study effect [40]. Egger’s test was used to assess the symmetry of the funnel plot [41].

To account for both the markedly effective rate and neurological deficits, we used multivariate methods to determine the dependency between outcomes. Clustering methods and 2-dimensional plots were used to produce clusters of treatments [42]. Using the clusterank command, clustered ranking plots can be obtained using the STATA program. The markedly effective rate and neurological deficits became the data variable containing the SUCRA scores for all treatments in this network. The different colors correspond to the estimated clusters and were utilized for grouping the treatments according to their similarity for both outcomes.

## Results

### Literature search and characteristics of the included studies

**Fig. 1:**
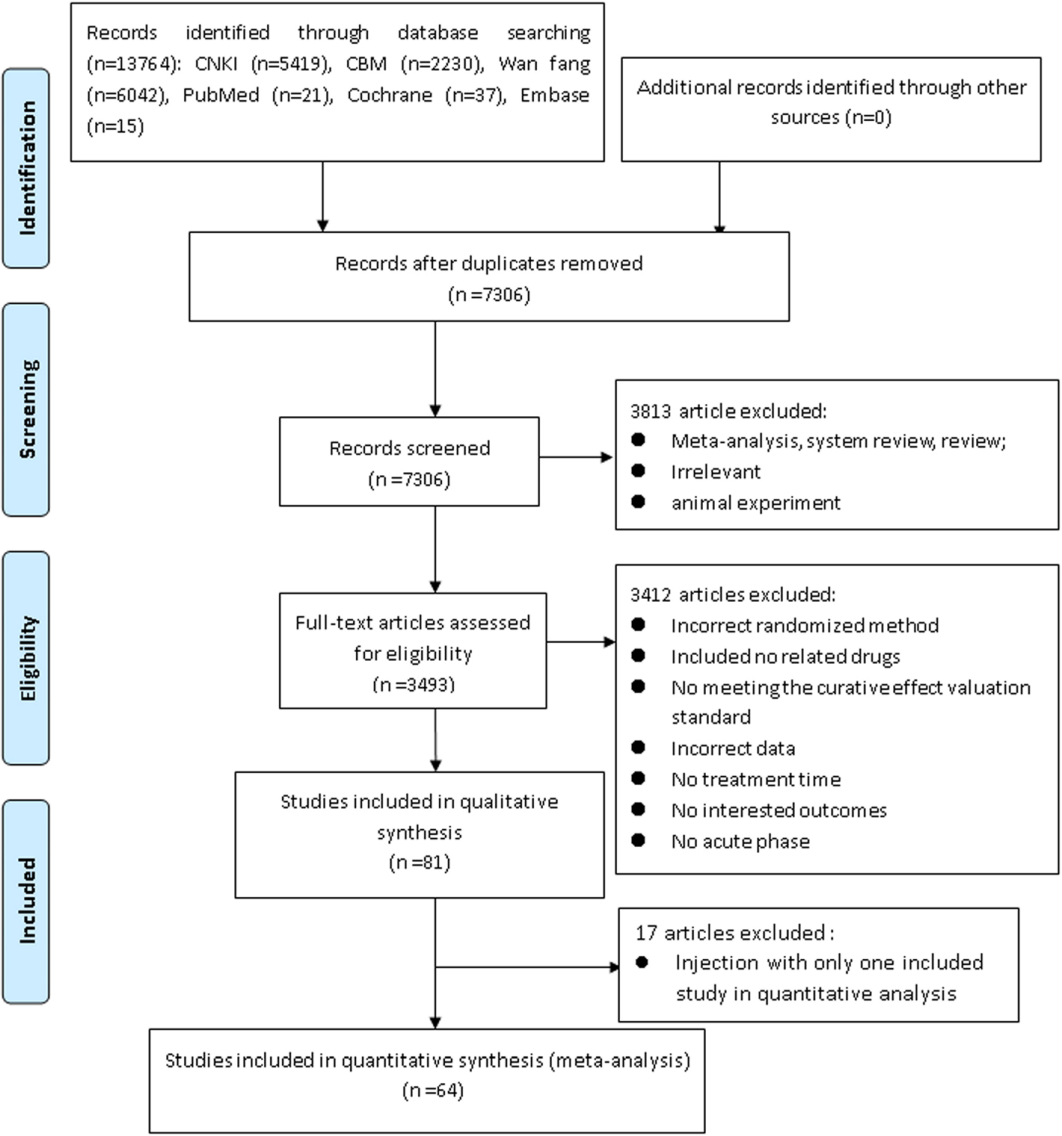
Flow diagram of the study search

Figure 1 shows the PRISMA flow diagram. A total of 13,764 articles were identified from electronic databases. After the exclusion of duplications, reviews, and obviously irrelevant studies by reading titles and abstracts, 3493 papers were downloaded for additional review. A total of 3429 RCTs were excluded for the following reasons: non-RCTs, non-acute phase, not meeting intervention and the curative effect valuation standard, incorrect data, no treatment time, and no outcomes of interest. Hence, 64 studies and 15 CHIs were included in the NMA. All studies were published in Chinese from 2006 to 2015.

### Characteristics of the included studies

**Fig. 2:**
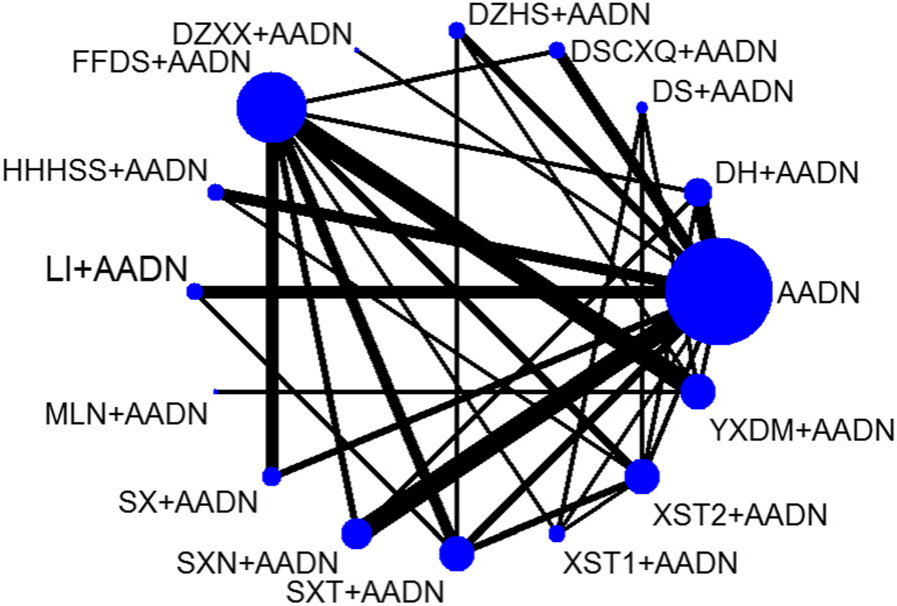
Network of the included comparisons. Note: Nodes are proportional to the number of patients included in the corresponding treatments, and edges are weighted according to the number of studies included in the respective comparisons

The 64 RCTs [28, 43–105] included 6225 participants, with sample sizes varying from 26 to 300 participants. All RCTs were conducted among Chinese populations in China. All participants were evaluated using cranial CT or NMRI. The rang of participants was approximately 35 to 87 years. There were more male patients (59.4%) than females. This study included 16 treatments for ACI: AADN, Shuxuening injection(SXN) + AADN, Shuxuetong injection (SXT) + AADN, Shenxiong injection (SX) + AADN, Mailuoning injection (MLN) + AADN, Honghuahuangsesu injection (HHHSS) + AADN, Fufangdanshen injection (FFDS) + AADN, Dengzhanhuasu injection (DZHS) + AADN, Dengzhanxixin injection (DZXX) + AADN, Danshenchuanxiongqin injection (DSCXQ) + AADN, Danshen injection (DS) + AADN, Danhong injection (DH) + AADN, Yinxingdamo injection (YXDM) + AADN, Ligustrazine injection (LI) + AADN, Xuesaitong injection (XST2) + AADN, and Xueshuantong injection (XST1) + AADN; to concisely express the results of this research, we used the abbreviations of the TCM injections to replace the treatments. The treatment abbreviations are shown in Table 1. The acute phase was no more than 30 days, with 62.5% of the cases having an acute phase of less than 72 h. The duration of treatment for both the experimental and control groups was no more than 30 days. Figure 2 shows a network graph comparing fifteen CHIs. There were 120 pairwise comparisons including 40 direct comparisons. Table 2 provides a summary of the included studies. Additional file 3 showed the more details of included CHIs.

**Table 1:** Treatment abbreviations

Full name	Abbreviations
Aspirin + Anticoagulants + Dehydrant + Neuroprotectant	AADN
Ligustrazine injection	LI
Xueshuantong injection	XST1
Xuesaitong injection	XST2
Shuxuening injection	SXN
Dengzhanxixin injection	DZXX
Dengzhanhuasu injection	DZHS
Shuxuetong injection	SXT
Danhong injection	DH
Fufangdanshen injection	FFDS
Yinxingdamo injection	YXDM
Mailuoning injection	MLN
Honghuahuangsesu injection	HHHSS
Shenxiong glucose injection	SX
Danshen Chuanxiongqin injection	DSCXQ
Danshen injection	DS

**Table 2:** Characteristics of the studies included in this meta-analysis

Study	Acute phase	Sex (M/F)	Age	Experimental group			Control group			Course	Outcomes	ADRs/ADEs
				N	T1	Dosage	N	T2	Dosage			
Yu YC 2014	72 h	38/30	62.5 (44–76)	34	XST1	500 mg	34	AADN	–	14d	(1)	None
Yu YM 2009	72 h	71/45	62.5 (40–72)	58	DH	20 ml	58	FFDS	20 ml	15d	(1)(2)	Unclear
Yu Y 2015	72 h	35/21	54.3 (38–71)	28	XST1	500 mg	28	FFDS	20 ml	14d	(1)(2)	6
Zhang ZJ 2008	74 h	41/30	64.3	35	SX	100 ml	36	FFDS	20 ml	14d	(1)	None
Zheng XD 2007	72 h	39/21	66.4	30	YXDM	20 mL	30	FFDS	30 ml	14d	(1)	None
Zhou SJ 2011	1 M	46/34	62 (42–75)	40	SX	100 ml	40	AADN	–	14d	(1)(2)	None
Zhou SF 2009	48 h	52/42	65.6 (62–86)	48	SX	200 ml	46	FFDS	20 mL	21d	(1)(2)	10
Zhou SH 2013	72 h	50/40	62	45	SXN	20 ml	45	AADN	–	14d	(1)(4)	None
Xu XY 2011	12 h	40/20	55.9 (45–73)	30	YXDM	20 ml	30	DS	20 ml	21d	(1)(2)(3)	Unclear
Xie S 2011	24 h	42/30	61.4 (53–78)	36	SXT	6 ml	36	FFDS	20 ml	14d	(1)	None
Xie YG 2010	48 h	39/31	61.6 (50–76)	35	SXT	6 ml	35	DZHS	50 mg	14d	(1)(2)	None
Xu LL 2011	72 h	48/34	63.0 (55–81)	42	LI	120 mg	42	AADN	–	14d	(1)(2)	Unclear
Xu XQ 2012	72 h	64/44	57.9	54	YXDM	20 ml	54	FFDS	20 ml	14d	(1)(2)	1
Yang HJ 2007	24 h	50/34	53.6 (39–75)	48	DSCXQ	10 ml	36	FFDS	10 ml	30d	(1)(2)	1
Yang YF 2012	48 h	–	68	112	DH	30 ml	101	AADN	–	21d	(1)(2)	Unclear
Yao QY 2010	72 h	64/40	70.8 (54–82)	56	SXT	6 ml	48	LI	12 ml	14d	(1)(2)	2
Yao J 2010	72 h	37/27	60.5 (49–76)	32	YXDM	20 ml	32	DZHS	50 mg	14d	(1)(2)	None
Xie RP 2010	72 h	52/28	42–80	40	SXN	20 ml	40	FFDS	20 mL	15d	(1)	None
Tan SY 2016	72 h	49/37	39–82	43	DH	30 ml	43	AADN	–	14d	(1)(2)	None
Wang WP 2015	72 h	49/41	68.2 (60–78)	45	SXN	20 mL	45	FFDS	20 mL	14d	(1)(2)	Unclear
Ren HM 2009	72 h	38/26	40–78	32	SX	100 ml	32	AADN	–	14d	(1)(2)	None
Sun HJ 2013	72 h	42/36	60.9 (40–70)	39	LI	120 mg	39	AADN	–	14d	(1)(2)	Unclear
Tang JP 2013	72 h	47/45	51.8 (43–73)	46	HHHSS	100 mg	46	AADN	–	14d	(1)(2)	None
Tang FY 2009	72 h	39/33	64.0 (50–78)	36	DZHS	50 mg	36	AADN	–	14d	(1)(2)	None
Tang XJ 2013	72 h	45/33	58	39	YXDM	20 mg	39	AADN	–	14d	(1)(2)	None
Wang L 2010	48 h	26/22	65.6 (62–86)	40	SX	200 mL	40	FFDS	20 mL	21d	(1)(2)	Unclear
Wang YL 2013	168 h	33/23	32–88	28	SXT	6 ml	28	DZHS	40 ml	14d	(2)	Unclear
Lan Y 2015	72 h	41/39	54–72	40	DSCXQ	10 ml	40	AADN	–	14d	(1)(2)	3
Li M 2014	24 h	63/17	48–76	40	DH	30 ml	40	AADN	–	14d	(2)	Unclear
Liu YP 2010	72 h	45/35	51–71	40	DH	40 ml	40	AADN	–	15d	(1)	1
Ma J 2010	24 h	29/31	59.1 (50–68)	30	DZHS	20 mg	30	AADN	–	14d	(1)(2)	Unclear
Ma J 2015	72 h	49/41	65.5 (45–79)	45	DH	20 ml	45	AADN	–	28d	(1)(4)	Unclear
Chen H 2015	24 h	74/60	59.4 (46–87)	67	SXT	6 ml	67	AADN	–	14d	(1)(2)	5
Luan T 2014	48 h	52/43	57.5	50	XST2	0.4 g	45	DS	20 ml	15d	(1)(4)	7
Huang ML 2012	72 h	158/142	62.6 (42–71)	150	XST1	500 mg	150	DS	25 ml	14d	(1)	1
Li X 2015	72 h	129/71	62.9	100	HHHSS	0.1 g	100	AADN	–	14d	(1)(2)	Unclear
Ma ZL 2011	72 h	74/46	62.8 (45–84)	60	HHHSS	0.1 g	60	AADN	–	14d	(1)(2)	None
Zhang Y 2012	48 h	52/28	57.5 (35–80)	40	DSCXQ	100 mg	40	AADN	–	14d	(1)(2)	1
Liu M 2014	48 h	79/57	60.2 (40–76)	68	DSCXQ	10 mL	68	AADN	–	14d	(1)(2)	2
Chen YC 2011	72 h	44/24	57.9	34	YXDM	20 ml	34	FFDS	20 ml	14d	(1)(2)	1
Yang RF 2013	48 h	52/48	60.8 (30–83)	50	SXT	6 g	50	AADN	–	14d	(1)	Unclear
Lin YF 2008	72 h	39/21	66.5	30	YXDM	20 ml	30	FFDS	20 ml	14d	(1)(3)	None
Peng T 2006	48 h	51/48	68	49	SXT	7 ml	50	FFDS	20 ml	14d	(1)(3)	1
Li XH 2011	168 h	–	63.5(40–78)	120	SXN	20 ml	120	AADN	–	14d	(1)(2)	Unclear
Liu L 2015	6-72 h	74/46	61.4 (39–76)	60	DH	20 ml	60	SXN	20 ml	14d	(1)	None
Zhang YH 2010	6-72 h	62/34	58.9 (39–81)	48	DH	30 ml	48	XST2	400 mg	14d	(1)(2)	5
Fan J 2006	72 h	56/32	64 (41–82)	44	YXDM	20 ml	44	FFDS	20 ml	14d	(2)	Unclear
Liu HY 2014	96 h	67/57	61.9 (38–83)	64	XST2	0.4 g	60	FFDS	30 ml	15d	(1)	None
Li FQ 2010	72 h	74/46	64	60	YXDM	30 ml	60	FFDS	40 ml	15d	(1)	Unclear
Lian CL 2013	360 h	49/43	62 (43–77)	46	LI	120 mg	46	AADN	–	30d	(1)	None
Chen S 2006	72 h	81/53	67.9	70	XST2	800 mg	64	XST1	10 ml	14d	(1)(2)	None
Cao LZ 2012	72 h	71/9	59.3	40	XST2	500 mg	40	FFDS	1.0 g	14d	(1)(2)	Unclear
Liu Y 2009	72 h	79/43	65.1 (43–73)	62	SXT	6 ml	60	XST2	8 ml	14d	(1)	1
Luo XD 2011	72 h	35/25	61.8	30	XST2	0.4 g	30	AADN	–	14d	(1)(2)	3
Liao MJ 2014	6-72 h	43/17	62.5 (36–80)	30	HHHSS	100 ml	30	XST2	400 mg	14d	(1)	Unclear
Shi JL 2010	72 h	51/33	62	42	YXDM	20 ml	42	LI	200 mg	30d	(2)	Unclear
Ni H 2010	24 h	61/55	62.7 (54–74)	59	SXN	20 ml	57	AADN	–	14d	(1)	Unclear
Li CP 2007	8-72 h	106/54	63.8 (42–85)	80	SXN	10-20 ml	80	AADN	–	14d	(1)	Unclear
Zhou ZP 2011	24 h	48/33	62.2	42	DZXX	30 ml	39	AADN	–	15d	(1)	Unclear
Chen C 2015	72 h	37/31	67.5	34	SXT	6 ml	34	XST2	400 mg	14d	(1)(2)	14
Chen JY 2012	72 h	73/35	67 (40–76)	54	SXT	6 ml	54	FFDS	20 ml	14d	(1)(2)	Unclear
Luo QY 2007	72 h	54/36	63.6 (37–81)	45	SXN	6 ml	45	AADN	20 ml	14d	(1)	None
Chen J 2007	72 h	45/35	61	40	YXDM	20 ml	40	MLN	20 ml	15d	(1)(2)(3)	Unclear
Zhang XL 2005	6d	37/13	35–80	26	MLN	20 ml	24	FFDS	20 m l	21d	(2)	None

*M* male, *F* female, *AVG* average, *E* experimental group, *C* control group, *W* week, *D* day, *AADN* aspirin + anticoagulants + dehydrant + neuroprotectant, *DH* Danhong injection + AADN, *DS* Danshen injection + AADN, *DSCXQ* Danshenchuangxiongqin injection + AADN, *DZHS* Dengzhanhuasu injection + AADN, *DZXX* Dengzhanxixin injection + AADN, *FFDS* Fufangdanshen injection + AADN, *HHHSS* Honghuahuangsesu injection + AADN, *SX* Shenxiong glucose injection + AADN, *SXT* Shuxuetong injection + AADN, *XST1* Xueshuantong injection + AADN, *XST2* Xuesetong injection + AADN, *LI* Ligustrazine injection + AADN, *YXDM* Yinxingdamo injection + AADN, *SXN* Shuxuening injection + AADN, *MLN* Mailuoning injection + AADN, *ADRs* adverse drug reactions, *ADEs* adverse drug events; (1): markedly effective rate; (2): neurological impairment; (3): death; (4): activities of daily living function; N: sample size; T1: therapy of experiment; T2: Therapy of control; N:Number of studies; −: No report

### Quality of the included studies

We used the Cochrane Handbook version 5.1.0 and CONSORT statement to conduct a quality evaluation of the included studies. All studies mentioned the use of random distribution, while ten studies [44, 64, 68, 73, 75, 82, 95–97, 101] described a satisfactory method of randomization including random number tables or the envelope method. Two studies [60, 85] reported information about blinding. All studies provided information on patients who were lost to follow-up or dropped out. All studies reported the eligibility criteria for participants and statistical methods. Approximately 74.6% of the studies provided information about adverse events. Details on risk of bias are shown in Additional file 4: Figure S1.

### Pairwise meta-analysis

#### Pairwise meta-analysis of the markedly effective rate

**Fig. 3:**
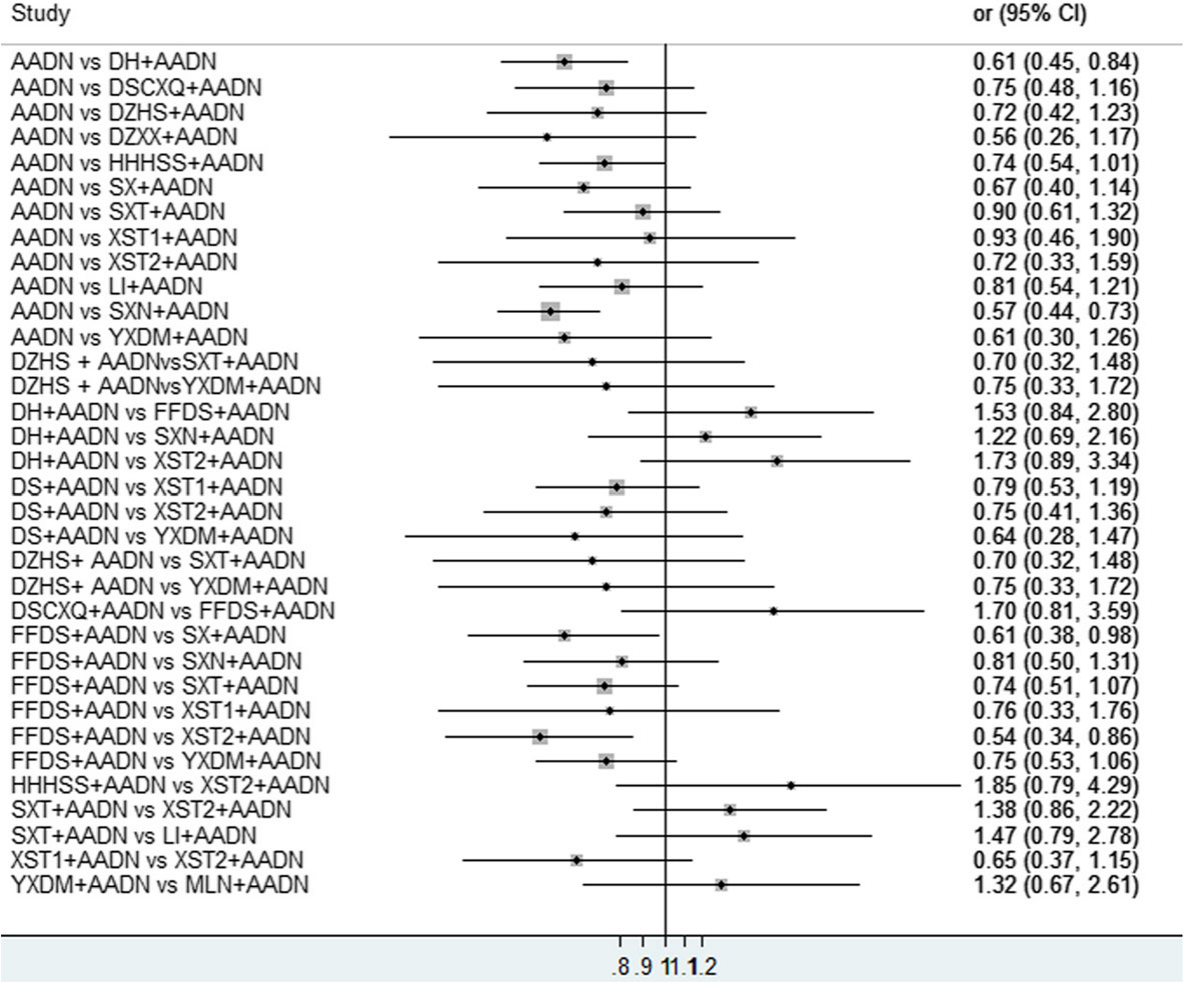
Forest graph of Meta-analysis on the markedly effective rate

Fifty-nine RCTs reported markedly effective rates; in these RCTs, 5864 patients were involved and 34 regimens were included. Table 3 summarizes the results of the pairwise meta-analysis regarding the markedly effective rates. There was no significant heterogeneity in the pooled analysis of all included studies (*P* > 0.1; *I* ^*2*^ < 50%); the results of the heterogeneity test are shown in Table 2. The direct comparison showed that DH and SXN were more beneficial in improving the markedly effective rate than AADN (AADN versus DH: *OR* = 0.61, *95% CI* = 0.45–0.84; versus SXN: *OR* = 0.57, *95% CI* = 0.44–0.73). SX and XST2 were more beneficial than FFDS (FFDS versus SX: *OR* = 0.61, *95% CI* = 0.38–0.98; versus XST2: *OR* = 0.54, *95% CI* = 0.34–0.86). Other treatment comparisons failed to reach statistical significance (the *95% CI* included 1). The detailed results are shown in Fig. 3.

**Table 3:** A summary of the meta-analysis for the markedly effective rate

AADN	**0.61(0.45,0.84)** ***P*** **= 0.96** ^***i2***^ **= 0%**		0.75(0.48,1.16) *P* = 0.97 ***l***^*2*^ = 0%	0.72(0.42,1.23) *P* = 0.82 *I*^*2*^ = 0%	0.56(0.26,1.17)	–	0.74(0.54,1.01) *P* = 0.91 *I*^*2*^ = 0%	0.67(0.40,1.14) *P* = 0.92 *I*^*2*^ = 0%	0.90(0.61,1.32) *P* = 0.71 *I*^*2*^ = 0%	0.93(0.46, 1.90)	0.72(0.33, 1.59)	0.81(0.54, 1.21) *P* = 0.79* I*^*2*^ = 0%	0.61(0.30,1.26)	**0.57(0.44,0.73)** *P* = 0.19 *I*^*2*^ = 35.4%	**–**
**3.89** **(2.26, 6.26)**	DH	–	–	–	–	1.53 (0.84,2.80)	–	–	–	–	1.73 (0.89, 3.34)	–	–	1.22 (0.69,2.16)	–
0.8 (0.29, 1.8)	0.22 (0.07,0.52)	DS	–	–	–	–	–	–	–	0.79 (0.53,1.19)	0.75 (0.41, 1.36)	–	0.64 (0.28,1.47)	–	–
**2.14** **(1.02, 3.99)**	0.59 (0.23,1.23)	3.3 (0.91,8.58)	DSCXQ	–	–	1.70 (0.81,3.59)	–	–	–	–	–	–	–	–	–
2.22 (0.95, 4.47)	0.61 (0.22,1.35)	3.38 (0.92,8.96)	1.16 (0.37,2.82)	DZHS	–	–	–	–	0.70 (0.32,1.48)	–	–	–	0.75 (0.33,1.72)	–	
**5.36** **(1.06,16.68)**	1.47 (0.26,4.87)	**8.32** **(1.12,30.55)**	2.82 (0.44,9.73)	2.82 (0.42,9.88)	DZXX	–	–	–	–	–	–	–	–	–	–
0.94 (0.57, 1.46)	0.25 (0.13,0.44)	1.41 (0.51,3.11)	0.49 (0.21,0.96)	0.48 (0.19,1.01)	**0.29** **(0.05,0.93)**	FFDS	–	**0.61(0.38, 0.98)** ***P*** **= 0.89 ***I*^***2***^ **= 0%**	0.74(0.51,1.07)*P* = 0.86* I*^*2*^ = 0%	0.76(0.33, 1.76)	**0.54(0.34,0.86)*P*** **= 0.70 ** *I*^***2***^ **= 0%**	–	0.75(0.53,1.06)*P* = 0.98 *I*^*2*^ = 0%	0.81(0.50,1.31)*P* = 0.65 *I*^*2*^ = 0%	–
**3.34** **(1.66, 6.14)**	0.91 (0.37,1.91)	**5.14** **(1.48,13.28)**	1.75 (0.62,4)	1.75 (0.56,4.22)	1.02 (0.16,3.51)	**3.76** **(1.61,7.65)**	HHHSS	–	–	–	1.85 (0.79, 4.29)	–	–	–	–
**2.9** **(1.36, 5.46)**	0.79 (0.32,1.65)	**4.43** **(1.29,11.37)**	1.52 (0.53,3.47)	1.51 (0.49,3.6)	0.89 (0.14,3.03)	**3.18** **(1.56,5.83)**	0.97 (0.34,2.21)	SX	–	–	–	–	–	–	–
**3.27** **(1.86, 5.35)**	0.89 (0.42,1.66)	**4.96** **(1.68,11.54)**	1.71 (0.68,3.58)	1.68 (0.67,3.51)	1 (0.17,3.27)	**3.6** **(2.03,5.97)**	1.09 (0.44,2.23)	1.25 (0.52,2.55)	SXT	–	1.38(0.86, 2.22) *P* = 0.86 *I*^*2*^ = 0%	1.47 (0.79, 2.78)	–	–	–
1.27 (0.53, 2.6)	0.34 (0.13,0.76)	1.78 (0.7,3.81)	0.66 (0.21,1.62)	0.66 (0.2,1.63)	0.39 (0.06,1.38)	1.39 (0.59,2.84)	0.42 (0.14,1)	0.49 (0.16,1.16)	0.41 (0.16,0.87)	XST1	0.65 (0.37, 1.15)	–	–	–	–
**2.24** **(1.23, 3.77)**	0.61 (0.29,1.12)	**3.31** **(1.25,7.23)**	1.17 (0.46,2.51)	1.16 (0.43,2.56)	0.68 (0.12,2.28)	**2.47** **(1.36,4.14)**	0.74 (0.31,1.5)	0.86 (0.35,1.77)	0.71 (0.38,1.25)	1.99 (0.85,3.94)	XST2	–	–	–	–
1.64 (0.78, 3.09)	0.45 (0.18,0.96)	2.54 (0.7,6.6)	0.86 (0.29,2.01)	0.86 (0.27,2.06)	0.5 (0.08,1.73)	1.85 (0.76,3.8)	0.55 (0.19,1.25)	0.64 (0.22,1.48)	0.53 (0.22,1.07)	1.52 (0.47,3.66)	0.79 (0.31,1.68)	LI	–	–	–
**2.99** **(1.53, 5.34)**	0.81 (0.36,1.6)	**4.43** **(1.57,10.1)**	1.56 (0.59,3.4)	1.52 (0.59,3.24)	0.91 (0.15,3.1)	**3.24** **(1.85,5.34)**	1 (0.37,2.17)	1.14 (0.46,2.36)	0.96 (0.45,1.82)	**2.69** **(1.01,5.84)**	1.41 (0.65,2.69)	2.05 (0.74,4.58)	YXDM	–	1.32 (0.67,2.61)
**3.3** **(2, 5.14)**	0.9 (0.46,1.59)	**5.09** **(1.64,12.18)**	1.73 (0.71,3.61)	1.73 (0.64,3.79)	1.01 (0.18,3.3)	**3.69** **(1.97,6.31)**	1.1 (0.45,2.25)	1.28 (0.53,2.61)	1.08 (0.52,1.98)	**3.04** **(1.12,6.65)**	1.59 (0.75,2.97)	2.27 (0.91,4.75)	1.21 (0.54,2.33)	SXN	–
1.33 (0.2, 4.65)	0.36 (0.05,1.3)	1.98 (0.24,7.46)	0.69 (0.09,2.61)	0.68 (0.09,2.47)	0.41 (0.03,1.84)	1.45 (0.23,4.95)	0.44 (0.06,1.65)	0.51 (0.07,1.86)	0.43 (0.06,1.52)	1.2 (0.15,4.48)	0.63 (0.09,2.24)	0.91 (0.11,3.45)	0.45 (0.08,1.44)	0.42 (0.06,1.51)	MLN

The upper right corner is the Meta-analysis results. The bottom left corner is the network Meta-analysis results. Results are the odds ratios (*OR*) and related 95% credibility interval (*95% CI*) in the row-defining treatment compared with the column -defining treatment. *OR* higher than 1 favor the row-defining treatment, and vice versa. Significant effects are printed in bold. *AADN* aspirin + anticoagulants + dehydrant + neuroprotectant, *DH* Danhong injection + AADN, *DS* Danshen injection + AADN, *DSCXQ* Danshenchuangxiongqin injection + AADN, *DZHS* Dengzhanhuasu injection + AADN, *DZXX* Dengzhanxixin injection + AADN, *FFDS* Fufangdanshen injection + AADN, *HHHSS* Honghuahuangsesu injection + AADN, *SX* Shenxiong glucose injection + AADN, *SXT* Shuxuetong injection + AADN, *XST1* Xueshuantong injection + AADN, *XST2* Xuesetong injection + AADN, *LI* Ligustrazine injection + AADN, *YXDM* Yinxingdamo injection + AADN, *SXN* Shuxuening injection + AADN, *MLN* Mailuoning injection + AAD

#### Pairwise meta-analysis of the improvement of neurological impairment

**Fig. 4:**
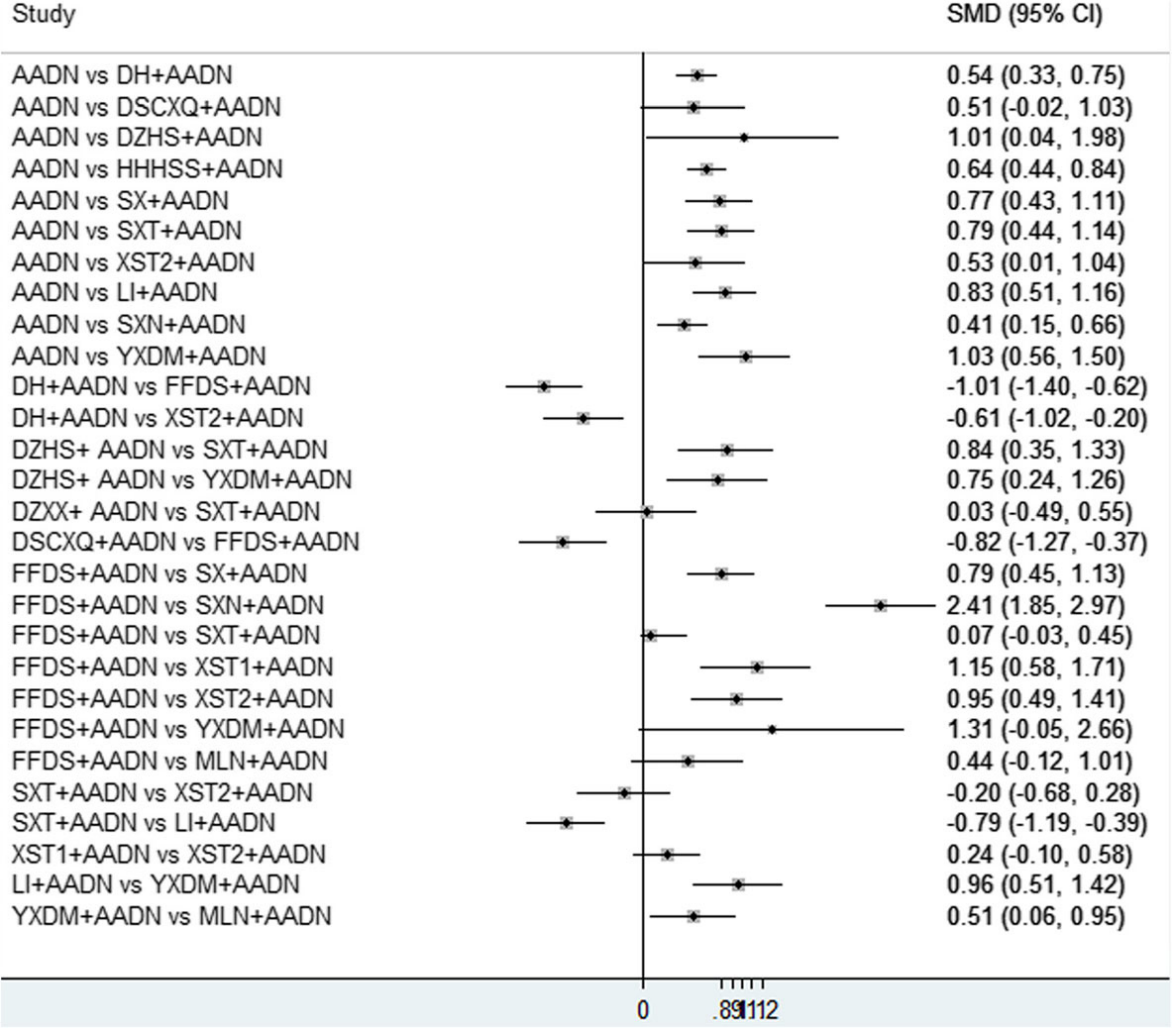
Forest graph of Meta-analysis on the neurological impairment

Forty-one RCTs reported an improvement of neurological impairment; in these RCTs, 3828 patients were involved and 29 regimens were included. When *P* was ≥0.1 and *I*^*2*^ was ≤50%, the Mantel-Haenszel fixed-effects model was used for the meta-analysis and vice versa. The results of the heterogeneity test are shown in Table 4. Table 4 summarizes the results of the pairwise meta-analysis regarding the improvement of neurological impairment. The direct comparison showed that DH, DZHS, HHHSS, SX, SXT, XST2, LI, YXDM, and SXN were more effective than AADN alone in the reduction of the neurological impairment score (AADN versus DH: *SMD* = 0.54, *95% CI* = 0.33–0.75; versus DZHS: *SMD* = 1.01, *95% CI* = 0.04–1.98; versus HHHSS: *SMD* = 0.64, *95% CI* = 0.44–0.84; versus SX: *SMD* = 0.77, *95% CI* = 0.43–1.11; versus SXT: *SMD* = 0.79, *95% CI* = 0.44–1.14; versus XST2: *SMD* = 0.53, *95% CI* = 0.01–1.04; versus LI: *SMD* = 0.83, *95% CI* = 0.51–1.16; versus YXDM: *SMD* = 1.03, *95% CI* = 0.56–1.50; versus SXN: *SMD* = 0.41, *95% CI* = 0.15–0.66). DH was more effective than FFDS (*SMD* = − 1.01, *95% CI* = − 1.40 to − 0.62) and XST2 (*SMD* = − 0.61, *95% CI* = − 1.02 to − 0.20). DSCXQ was more effective than FFDS (*SMD* = − 0.82, *95% CI* = − 1.27 to − 0.37). YXDM and SXT were more effective than DZHS (DZHS versus YXDM: *SMD* = 0.75, *95% CI* = 0.24–1.26; versus SXT: *SMD* = 0.84, *95% CI* = 0.35–1.33). SX, XST1, XST2, and SXN were more effective than FFDS (FFDS versus SX: *SMD* = 0.79, *95% CI* = 0.45–1.13; versus XST1: *SMD* = 1.15, *95% CI* = 0.58–1.71; versus XST2: *SMD* = 0.95, 95% *CI* = 0.49–1.41; versus SXN: *SMD* = 2.41, *95% CI* = 1.85–2.97). SXT was more effective than LI (*SMD* = − 0.79, *95% CI* = − 1.19 to − 0.39). YXDM was more effective than LI (LI versus YXDM: *SMD* = 0.96, *95% CI* = 0.51–1.42). MLN was more effective than YXDM (YXDM versus MLN: *SMD* = 0.51, *95% CI* = 0.06–0.95). Other treatment comparisons failed to reach statistical significance (the *95% CI* included 0). The detailed results are shown in Fig. 4.

**Table 4:** A summary of the meta-analysis for the improvement of neurological impairment

AADN	**0.54(0.33, 0.75)** ***I***^***2***^ **= 13.4%** ***P*** **= 0.32**	–	0.51(− 0.02, 1.03) ***I***^2^ = 79.8% *P* = 0.007	**1.01(0.04, 1.98)** ***I***^***2***^ **= 85.2%** ***P*** **= 0.009**	–	–	**0.64(0.44, 0.84)** ***I***^***2***^ **= 87.2%** ***P*** **= 0.000**	**0.77(0.43, 1.11)** ***I***^***2***^ **= 0%** ***P*** **= 0.60**	**0.79(0.44,1.14)**	–	**0.53(0.01, 1.04)**	**0.83** **(0.51, 1.16)** ***I***^***2***^ **= 80.4%** ***P*** **= 0.02**	**1.03(0.56, 1.50)**	**0.41(0.15, 0.66)**	–
− **0.71** **(− 1.28,-0.13)**	DH	–	–	–	–	**− 1.01** **(− 1.40,-0.62)**	–	–	–	–	**− 0.61** **(− 1.02,-0.20)**	–	–	–	
− 0.83 (− 2.21,0.56)	−0.12 (− 1.58,1.3)	DS	–	–	–	–	–	–	–	–	–	–	0.31 (− 0.20, 0.81)	–	–
− 0.52 (− 1.15,0.1)	0.19 (− 0.64,1.01)	0.31 (− 1.18,1.8)	DSCXQ	–	–	**− 0.82** **(− 1.27,-0.37)**	–	–	–	–	–	–	–	–	–
−0.59 (− 1.26,0.07)	0.12 (− 0.74,0.96)	0.24 (− 1.23,1.69)	−0.07 (− 0.96,0.8)	DZHS	–	–	–	–	**0.84** **(0.35, 1.33)**	–	–	–	**0.75** **(0.24, 1.26)**	–	–
−0.78 (− 2.19,0.63)	−0.07 (− 1.57,1.41)	0.05 (− 1.88,1.97)	−0.26 (− 1.78,1.2)	−0.19 (− 1.68,1.2)	DZXX	–	–	–	0.03 (− 0.49, 0.55)	–	–	–	–	–	–
0.22 (− 0.3,0.73)	**0.92** **(0.26,1.58)**	1.05 (− 0.32,2.4)	**0.74** **(0.01,1.47)**	**0.81** **(0.05,1.58)**	1.00 (− 0.43,2.43)	FFDS	–	**0.79** **(0.45,1.13)** ***I***^***2***^ **= 0%** ***P*** **= 1**	0.07 (−0.03, 0.45)	**1.15** **(0.58, 1.71)**	**0.95** **(0.49, 1.41)**	–	1.31 (−0.05,2.66) *I*^*2*^ = 95.7% P = 0	**2.41** **(1.85, 2.97)**	0.44 (−0.12, 1.01)
**−0.78** **(− 1.47,-0.09)**	− 0.07 (− 0.97,0.83)	0.05 (− 1.49,1.58)	−0.26 (− 1.19,0.6)	−0.19 (− 1.16,0.7)	0 (− 1.57,1.57)	**− 1** **(− 1.86,-0.13)**	HHHSS	–	–	–	–	–	–	–	–
− 0.67 (− 1.34,0)	0.04 (− 0.8,0.87)	0.16 (− 1.34,1.64)	−0.14 (− 1.03,0.7)	−0.08 (− 0.99,0.8)	0.11 (− 1.41,1.64)	**−0.88** **(− 1.55,-0.21)**	0.11 (− 0.85,1.08)	SX	–	–	–	–	–	–	–
**− 0.81** **(− 1.44,− 0.18)**	-0.1 (− 0.89,0.69)	0.02 (− 1.44,1.47)	−0.29 (− 1.14,0.5)	−0.22 (− 0.99,0.5)	−0.03 (− 1.29,1.23)	**−1.03** **(− 1.7,-0.36)**	−0.03 (− 0.97,0.91)	−0.14 (− 1,0.72)	SXT	–	− 0.20 (− 0.68,0.28)	**−**0.79 (− 1.19,-0.39)	–	–	–
− 0.6 (− 1.61,0.39)	0.11 (− 0.97,1.16)	0.23 (−1.41,1.85)	−0.08 (− 1.22,1.0)	−0.01 (− 1.16,1.1)	0.18 (− 1.48,1.83)	−0.82 (− 1.76,0.12)	0.18 (−1.05,1.39)	0.06 (− 1.06,1.18)	0.21 (− 0.87,1.28)	XST1	0.24 (− 0.10,0.58)	–	–	–	–
−0.56 (− 1.22,0.09)	0.14 (− 0.59,0.88)	0.27 (− 1.22,1.75)	−0.04 (− 0.92,0.8)	0.03 (− 0.85,0.9)	0.22 (−1.25,1.69)	**−0.78** **(− 1.44,-0.12)**	0.22 (− 0.74,1.17)	0.1 (− 0.77,0.97)	0.25 (− 0.5,1)	0.04 (− 0.88,0.97)	XST2	–	–	–	–
−0.47 (− 1.13,0.19)	0.24 (− 0.61,1.08)	0.36 (−1.09,1.82)	0.05 (− 0.84,0.9)	0.12 (− 0.74,0.9)	0.31 (− 1.16,1.79)	−0.69 (− 1.43,0.07)	0.31 (− 0.65,1.27)	0.2 (− 0.71,1.1)	0.34 (− 0.42,1.1)	0.13 (−1.01,1.28)	0.09 (− 0.78,0.96)	LI	**0.96** **(0.51, 1.42)**	–	–
**−1.14** **(− 1.74,-0.54)**	−0.43 (− 1.19,0.34)	−0.31 (− 1.57,0.94)	−0.61 (− 1.43,0.2)	−0.55 (− 1.3,0.22)	−0.36 (− 1.82,1.12)	**−1.35** **(− 1.89,-0.81)**	−0.36 (− 1.27,0.56)	−0.47 (− 1.28,0.33)	−0.33 (− 1.08,0.42)	−0.53 (− 1.59,0.52)	−0.57 (− 1.36,0.21)	−0.67 (− 1.42,0.08)	YXDM	–	**0.51** **(0.06,0.95)**
**−1.25** **(− 2.14,-0.37)**	− 0.54 (− 1.56,0.47)	−0.41 (− 2.01,1.18)	−0.72 (− 1.79,0.3)	−0.65 (− 1.73,0.4)	−0.46 (− 2.09,1.17)	**− 1.46** **(− 2.36,-0.58)**	−0.47 (− 1.6,0.65)	−0.58 (− 1.63,0.48)	−0.43 (− 1.48,0.59)	−0.64 (− 1.9,0.62)	−0.68 (− 1.73,0.36)	−0.77 (− 1.86,0.29)	−0.11 (− 1.1,0.88)	SXN	–
− 0.44 (− 1.45,0.57)	0.27 (− 0.84,1.37)	0.39 (− 1.16,1.94)	0.09 (− 1.06,1.2)	0.16 (− 0.98,1.2)	0.35 (− 1.32,2.02)	−0.65 (− 1.58,0.27)	0.34 (− 0.89,1.57)	0.23 (− 0.9,1.36)	0.37 (− 0.73,1.48)	0.17 (− 1.14,1.48)	0.13 (− 0.99,1.24)	0.03 (− 1.1,1.17)	0.7 (− 0.22,1.62)	0.81 (− 0.45,2.08)	MLN

The upper right corner is the Meta-analysis results. The bottom left corner is the network Meta-analysis results. Results are the *SMD* and related 95% credibility interval (*95% CI*) in the row-defining treatment compared with the column -defining treatment. SMD higher than 0 favor the column-defining treatment, and vice versa. Significant effects are printed in bold. *AADN* aspirin + anticoagulants + dehydrant + neuroprotectant, *DH* Danhong injection + AADN, *DS* Danshen injection + AADN, *DSCXQ* Danshenchuangxiongqin injection + AADN, *DZHS* Dengzhanhuasu injection + AADN, *DZXX* Dengzhanxixin injection + AADN, *FFDS* Fufangdanshen injection + AADN, *HHHSS* Honghuahuangsesu injection + AADN, *SX* Shenxiong glucose injection + AADN, *SXT* Shuxuetong injection + AADN, *XST1* Xueshuantong injection + AADN, *XST2* Xuesetong injection + AADN, *LI* Ligustrazine injection + AADN, *YXDM* Yinxingdamo injection + AADN, *SXN* Shuxuening injection + AADN, *MLN* Mailuoning injection + AADN

#### Pairwise meta-analysis of the death

Most studies did not report any deaths during the treatment period or during follow up after the treatment ended in all trials. Four studies [50, 81, 82, 102] reported no death during the treatment period. This result may mean that CHIs plus AADN is an effective approach in the treatment of ACI or short follow-up time.

#### Pairwise meta-analysis of the activities of daily living function

Three studies [46, 67, 71] assessed the activities of daily living function using the Barthel Index. Due to the limited quantity of the included studies, the Mantel-Haenszel random-effects model was used. There was a significant difference between the treatment group and the control group (SX versus AADN: *SMD* = 0.83, *95% CI* = 0.41–1.25; DSCXQ versus AADN: *SMD* = 0.73, *95% CI* = 0.28–1.18; DH versus AADN: *SMD* = 1.69, *95% CI* = 1.21–2.17).

### Results of the Bayesian network meta-analysis

In the original analysis, most studies did not mention the activities of daily living function or death from all causes within the treatment period or during the entire follow-up period. Therefore, the present study did not compare death or the activities of daily living function among different treatments; we only performed a NMA to compare the markedly effective rate and the improvement of neurological impairment among the different regimens of CHIs for ACI.

### Bayesian network meta-analysis of the markedly effective rate

According to the network of comparisons (Table 3), DH, DSCXQ, DZXX, HHHSS, SX, SXT, XST2, YXDM, and SXN improved the markedly effective rate more significantly than AADN alone (DH: *OR* = 3.89, *95% CI* = 2.26–6.26; DSCXQ: *OR* = 2.14, *95% CI* = 1.02–3.99; DZXX: *OR* = 5.36, *95% CI* = 1.06–16.68; HHHSS: *OR* = 3.34, *95% CI* = 1.66–6.14; SX: *OR* = 2.90, *95% CI* = 1.36–5.46; SXT: *OR* = 3.27, *95% CI* = 1.86–5.35; *XST2*: *OR* = 2.24, *95% CI* = 1.23–3.77; YXDM: *OR* = 2.99, *95% CI* = 1.53–5.34; SXN: *OR* = 3.3, *95% CI* = 2–5.14). Moreover, DZXX, HHHSS, SX, SXT, XST2, YXDM, and SXN were better than DS (DZXX: *OR* = 8.32, *95% CI* = 1.12–30.55; HHHSS: *OR* = 5.14, *95% CI* = 1.48–13.28; SX: *OR* = 4.43, *95% CI* = 1.29–11.37; SXT: *OR* = 4.96, *95% CI* = 1.68–11.54; XST2: *OR* = 3.31, *95% CI* = 1.25–7.23; YXDM: *OR* = 4.43, *95% CI* = 1.57–10.1; SXN: *OR* = 5.09, *95% CI* = 1.64–12.18). Additionally, HHHSS, SX, SXT, XST2, YXDM, and SXN were more effective than FFDS (HHHSS: *OR* = 3.76, *95% CI* = 1.61–7.65; SX: *OR* = 3.18, *95% CI* = 1.56–5.83; SXT: *OR* = 3.60, *95% CI* = 2.03–5.97; XST2: *OR* = 2.47, *95% CI* = 1.36–4.14; YXDM: *OR* = 3.24, *95% CI* = 1.85–5.34; SXN: *OR* = 3.69, *95% CI* = 1.97–6.31). YXDM and SXN were more effective than XST1 (YXDM: *OR* = 2.69, *95% CI* = 1.01–5.84; SXN: *OR* = 3.04, *95% CI* = 1.12–6.65).

Additional file 5: Figure S2 shows the inconsistency plot used to identify heterogeneity among studies in the closed loop of this NMA. Eleven triangular loops and 25 quadratic loops were present in the NMA; 83% of IF values with 95% CIs were truncated at zero, suggesting no significant inconsistency.

### Rank probability

**Fig. 5:**
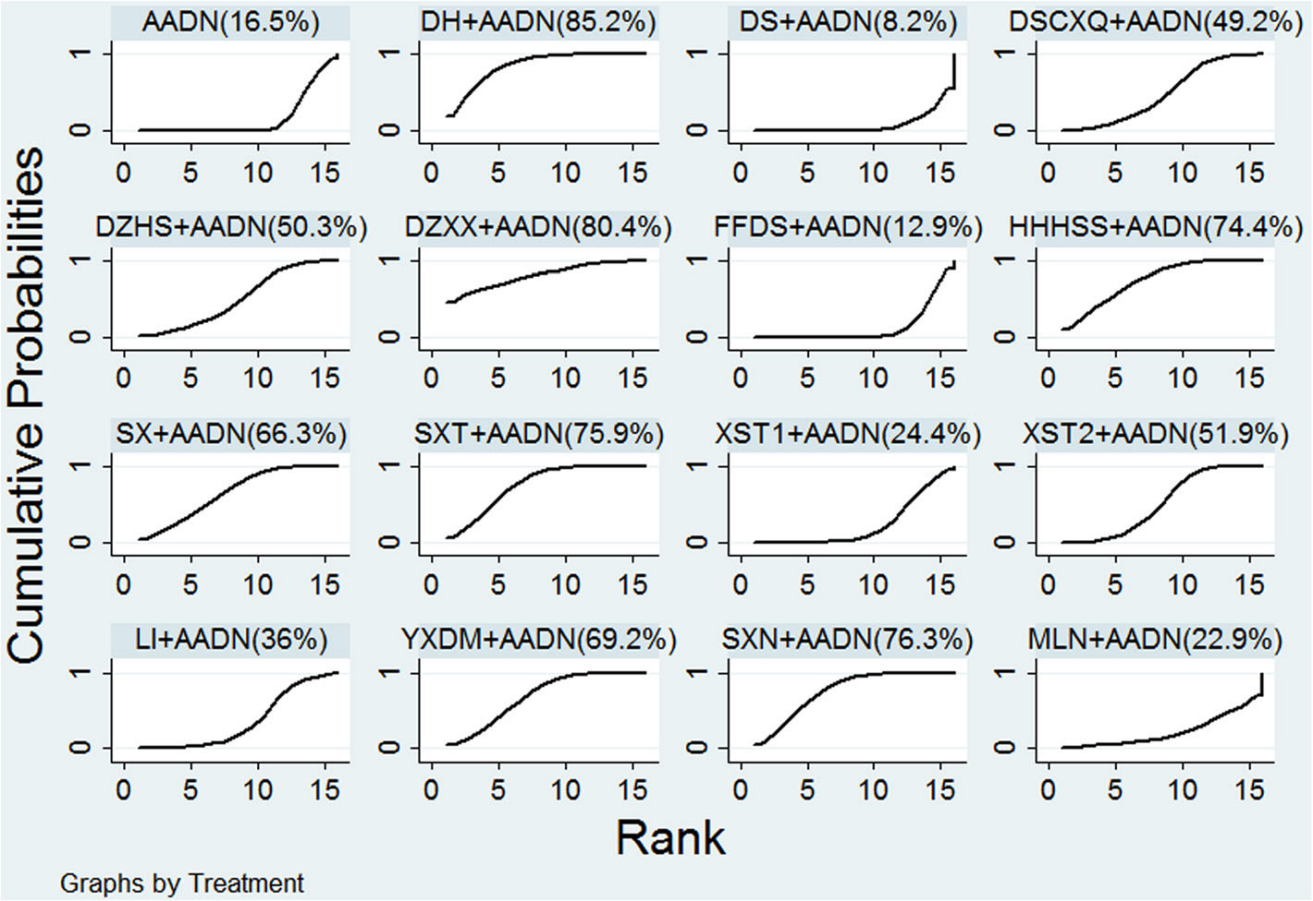
SUCRA for the markedly effective rate

Figure 5 shows the cumulative probabilities (SUCRA results) of CHIs that were the most effective when combined with AADN. DH had the highest likelihood of being the best treatment for the markedly effective rate (SUCRA-85.2%), followed by DZXX (SUCRA-80.4%), SXN (SUCRA-76.3%), SXT (SUCRA-75.9%), HHHSS (SUCRA-74.4%), YXDM (SUCRA-69.2%), SX (SUCRA-66.3%), XST2 (SUCRA-51.9%), DZHS (SUCRA-50.3%), DSCXQ (SUCRA-49.2%), LI (SUCRA-36.0%), XST1 (SUCRA-24.4%), MLN (SUCRA-22.9%), FFDS (SUCRA-12.9%), and DS (SUCRA-8.2%).

### Assessment of publication bias

The comparison-adjusted funnel plots (Additional file 6: Figure S3) for the markedly effective rate were asymmetric near the zero line. The result from Egger’s test was *P* = 0.047. Therefore, this study may have a small sample effect and publication bias.

### Bayesian network meta-analysis of the improvement of neurological impairment

According to the network of comparisons (Table 4), DH, HHHSS, SXT, YXDM, and SXN improved neurological impairment more significantly than AADN alone (DH: *SMD* = − 0.71, *95% CI* = − 1.28 to − 0.13; HHHSS: *SMD* = − 0.78, *95% CI* = − 1.47 to − 0.09; SXT: *SMD* = − 0.81, 95% CI = − 1.44 to − 0.18; YXDM: *SMD* = − 1.14, *95*% *CI* = − 1.74 to − 0.54; SXN: *SMD* = − 1.25, *95*% *CI* = − 2.14 to − 0.37). Moreover, DH, DSCXQ, DZHS, HHHSS, SX, SXT, XST2, YXDM, and SXN were more effective than FFDS (FFDS versus DH: *SMD* = 0.92, *95*% *CI* = 0.26–1.58; versus DSCXQ: *SMD* = 0.74, *95% CI* = 0.01–1.47; versus DZHS: *SMD* = 0.81, *95% CI* = 0.05–1.58; HHHSS: *SMD* = − 1, *95% CI* = − 1.86 to − 0.13; SX: *SMD* = − 0.88, *95% CI* = − 1.55 to − 0.21; SXT: *SMD* = − 1.03, *95% CI* = − 1.7 to − 0.36; XST2: *SMD* = − 0.78, *95% CI* = − 1.44 to − 0.12; YXDM: *SMD* = − 1.35, *95% CI* = − 1.89 to − 0.81; SXN: *SMD* = − 1.46, *95% CI* = − 2.36 to − 0.58). There was no statistical significance in other treatment comparisons.

As shown in Additional file 7: Figure S4, 9 triangular loops and seventeen quadratic loops were present in the NMA; 81% of IF values with 95% CIs were truncated at zero, suggesting no significant inconsistency.

### Rank probability

**Fig. 6:**
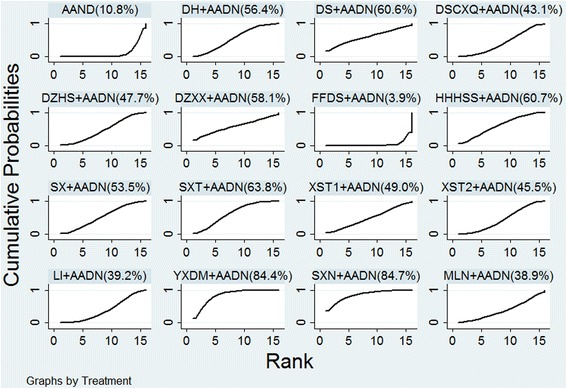
SUCRA for the improvement of neurological impairment

The cumulative probability analysis (SUCRA results) showed that SXN + AADN had the highest likelihood of improving the neurological impairment scores (SUCRA-84.7%), followed by YXDM (SUCRA-84.4%), SXT (SUCRA-63.8%), HHHSS (SUCRA-60.7%), DS (SUCRA-60.6%), DZXX (SUCRA-58.1%), DH (SUCRA-56.3%), SX (SUCRA-53.2%), XST1 (SUCRA-49.0%), DZHS (SUCRA-47.7%), XST2 (SUCRA-45.5%), DSCXQ (SUCRA-43.1%), LI (SUCRA-39.2%), MLN (SUCRA-38.9%), and FFDS (SUCRA-3.9%). The results are shown in Fig. 6.

### Simultaneous ranking of the interventions for two outcomes

**Fig. 7:**
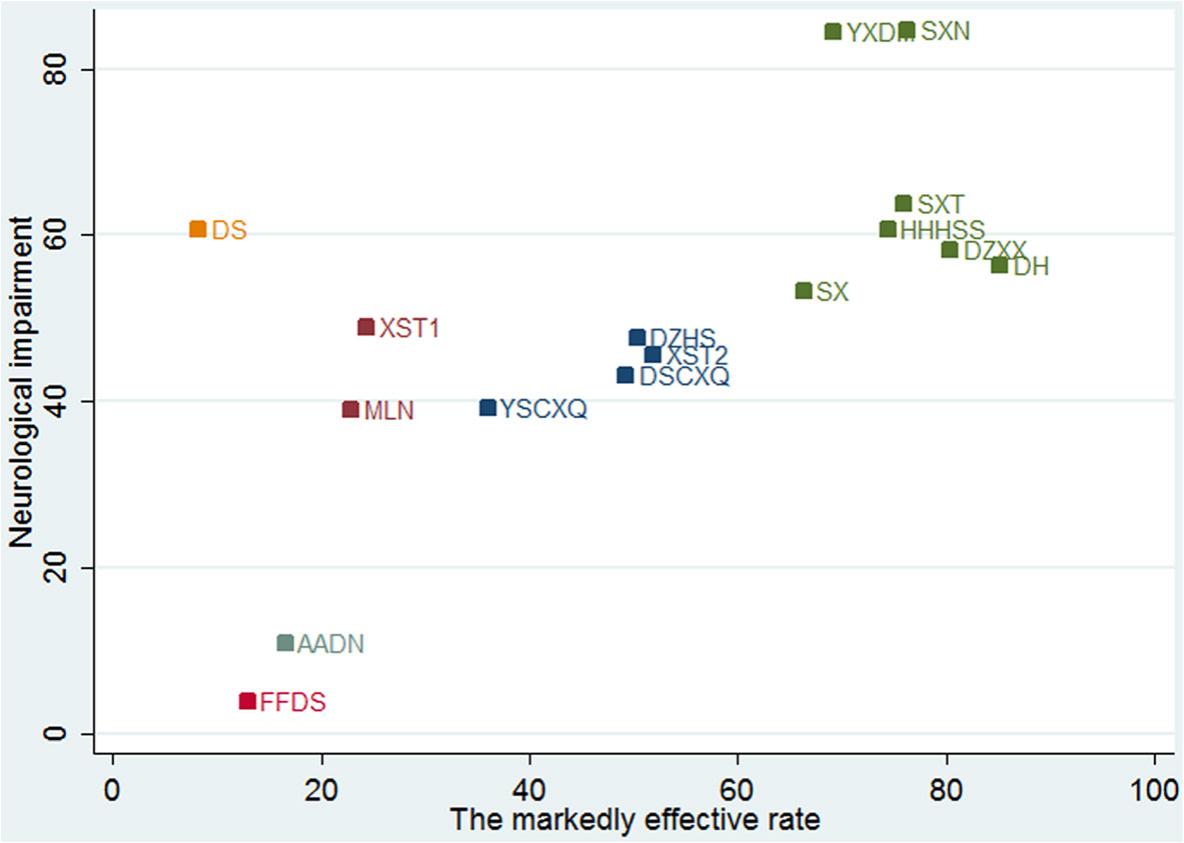
Clustered ranking plot of the networks

Clustered ranking plots of the network for the markedly effective rate and the improvement of neurological impairment score are shown in Fig. 7. Each color represents a group of treatments that belong to the same cluster. Treatments lying in the upper right corner are more effective than the other treatments. The upper right corner in Fig. 7 shows that SXN, YXDM, DH, SXT, HHHSS, DZXX and SX produce significantly better outcomes in ACI patients.

## Discussion

Considerable evidence exists regarding the clinical effectiveness of CHIs in ACI patients. Some CHIs have been widely used to strengthen clinical effectiveness, reduce neurological impairments and improve the patient’s quality of life. However, the majority of these findings have not been analyzed in head-to-head comparisons. Clinicians must decide among several therapeutic options for ACI patients. To address the absence of comparative data, we conducted an NMA to comprehensively estimate the effectiveness of different CHIs combined with AADN for ACI.

This NMA consisted of 64 RCTs that included 6225 participants; fifteen CHIs were identified in the treatment of ACI, including injections of SXN, SXT, SX, MLN, HHHSS, FFDS, DZHS, DZXX, DSCXQ, DS, DH, YXDM, LI, XST1, and XST2. In terms of the improvement of the markedly effective rate, DH had the highest likelihood of being the best treatment in terms of the markedly effective rate. On account of improvement of neurological impairment, SXN had the highest probability of being the best treatment.

The clustered ranking according to two outcomes revealed that the markedly effective rate and the improvement of the neurological impairment cluster were best for SXN, YXDM, DH, SXT, HHHSS, DZXX and SX. SXN and YXDM are shown at the top right corner. Previous meta-analyses [106–108] found that SXN and YXDM as adjuvant treatments for ACI were beneficial compared to AADN alone. SXN and YXDM are *Ginkgo biloba* extracts (GBEs), both of which are extracted from *Ginkgo biloba* leaves. *Ginkgo biloba* leaves, the TCM for activating blood circulation, mainly contain ginkgo flavonoids, ginkgolides, and bilobalide and have been used as a therapeutic agent for managing cerebrovascular and neurological disorders [109, 110]. GBE exhibits a wide variety of biological activities, including anti-inflammation and antioxidant effects [111, 112]. ACI is the process whereby artery stenosis or blockage causes brain tissue hypoxic ischemia, resulting in brain dysfunction [6]. There is considerable evidence suggesting the active repair and recovery mechanisms following stroke, and neurogenesis is one of them [113]. GBE not only has antioxidant, anti-atherogenesis and angiogenic properties but can also strengthen repair and regeneration mechanisms and prevent cell death, protect the brain from further damage and improve neurological deficits following stroke [113–115]. The neuroprotective mechanism has been attributed to the heme oxygenase 1 (HO1)/Wnt canonical pathway as well as neuritogenic and angiogenic effects [113, 116]. HO1, a key component of the EGb 761 neuroprotective signaling pathway, activates the signaling pathway mechanisms of angiogenesis, cell survival and neuroplasticity, and neurogenesis [113]. Thus, GBE could enhance the post-stroke regeneration process to improve treatments for stroke recovery. Further research is desirable to shed more light on the mechanism underlying the effects of GBE on ACI.

A NMA was used to compare the effectiveness of different CHIs to identify the best CHIs for ACI. This study is the first indirect comparison using a network approach to compare the effectiveness of CHIs, which is valuable for clinicians selecting CHIs for ACI treatment. However, some limitations existed in this NMA.

First, all trials reported random distribution, while ten studies described the randomization methods including random number tables or the envelope method. Information about allocation concealment and blinding was not clear in the majority of trials and may have therefore affected the reliability of the results. Second, the systematic review included only published studies in the database, with no relevant gray literature, which likely caused a selection bias in the literature. Third, the study aimed to use a NMA to evaluate the clinical effectiveness of 37 CHIs combined with an AADN regimen; however, only 15 CHIs were included in the NMA. Thus, more rigorously designed RCTs focused on the 22 additional CHIs are needed to confirm the effectiveness of CHIs for ACI. Fourth, due to the original research limitation, we failed to evaluate the long-term effect of CHIs. Additionally, with the limited data extracted from the original research, we failed to evaluate the ability of CHIs to improve the activities of daily living function and reduce mortality. Fifth, our results might have limited generalizability because all of the included RCTs were conducted in China among Chinese populations. Therefore, it is uncertain whether the effect may change when CHIs are used in populations of other ethnicities and in different geographical locations. In addition, though acute phases were limited, the severities of patient were various. This point may influence the results. Sixth, a NMA compares multiple treatments by incorporating direct and indirect evidence into a general statistical framework. One issue with the validity of a NMA is the inconsistency between direct and indirect evidence. Hence, to improve the reliability of our results, we used a random-effects model within a Bayesian framework. Although, head-to-head trials provide the highest level of evidence when comparing interventions, the quantity of data for some CHI direct trials was small, such as DH versus FFDS, SXN, and XSTT. Large RCTs are needed to specifically compare CHIs with one another.

## Conclusions

In summary, our evidence suggested that DH injection plus AADN was the optimum treatment regimen for patients with ACI to improve the markedly effective rate. SXN injection plus AADN was the optimum treatment regimen for ACI to improve the neurological impairment score. Considering both the markedly effective rate and the improvement of neurological impairment, SXN, YXDM, DH, SXT, HHHSS, DZXX and SX plus AADN were the optimum treatment regimens for ACI, especially SXN + AADN and YXDM + AADN. In terms of limitations, highest levels of evidence need to support our conclusions.

## Additional files

Additional file 1: **Table S1.** List of search terms. (DOC 20 kb)

Additional file 2: Search strategy. (DOC 18 kb)

Additional file 3: More details about the product information of CHIs. (DOC 42 kb)

Additional file 4: **Figure S1.** Risk of bias summary. Note: Green: low risk of bias; Yellow: unclear risk of bias; Red: high risk of bias. (JPEG 1934 kb)

Additional file 5: **Figure S2.** Inconsistency test for the markedly effective rate. (JPEG 953 kb)

Additional file 6: **Figure S3.** Comparison adjusted funnel plot. (JPEG 533 kb)

Additional file 7: **Figure S4.** Inconsistency test for improvement of neurological impairment. (JPEG 693 kb)

## Abbreviations

AADN: Aspirin + anticoagulants + dehydrant + neuroprotectant
ACI: Acute cerebral infarction
CBM: Chinese biomedical literature database
CHIs: Chinese herbal injections
CNKI: China national knowledge infrastructure database
CI: Credible interval
CT: Computed tomography
DH: Danhong injection
DS: Danshen injection
DSCXQ: Danshenchuanxiongqin injection
DZHS: Dengzhanhuasu injection
DZXX: Dengzhanxixi injection
FFDS: Fufangdanshen injection
GBEs: *Ginkgo biloba* extracts
HHHSS: Honghuahuangsesu injection
IF: Inconsistency factor
LI: Ligustrazine injection
MeSH: Medical subject heading
MLN: Mailuoning injection
MRI: Magnetic resonance imaging
NMA: Network meta-analysis
ORs: Odds ratios
RCTs: Randomized controlled trials
SMDs: Standardized mean differences
SUCRA: Surface under the cumulative ranking probabilities
SX: Shenxiong injection
SXN: Shuxuening injection
SXT: Shuxuetong injection
TCM: traditional Chinese medicine
XST1: Xueshuantong injection
XST2: Xuesaitong injection
YXDM: Yinxingdamo injection
