# Supplementary material for: Comparative efficacy of Chinese herbal injections for treating acute cerebral infarction: a network meta-analysis of randomized controlled trials
Source: BMC Complement Altern Med. 2018 Apr 3;18:120. doi: 10.1186/s12906-018-2178-9 (PMC5883592; doi:10.1186/s12906-018-2178-9)
Supplement: Supplementary file 1 — Table S1. List of search terms. (DOC 20 kb) [file 12906_2018_2178_MOESM1_ESM.doc]

S1 Table. List of search terms.

| English search terms | Chinese search terms |
| --- | --- |
| Compound danshen injection OR Composite salvia miltiorrhiza injection OR Fufangdanshen injection OR Fufangdanshen zhusheye | 复方丹参注射液OR复方丹参注射剂OR香丹注射液OR香丹注射剂OR注射用复方丹参 |
| Safflor injection OR Honghua injection OR Honghua zhusheye | 红花注射液OR红花注射剂 |
| Tetramethylpyrazine injection OR Chuanxiongqin OR Chuanxiongqin zhusheye OR Chuanxiongqin injection | 川青OR瑞科林OR连通OR博盈康OR川芎嗪注射液OR川芎嗪注射剂OR注射用盐酸川芎嗪 |
| Sanqi Panax Notoginseng OR Xueshuantong Injection OR Xueshuantong zhusheye OR Shengtaixueshuantong injection | 血栓通注射液OR血栓通注射剂OR田七人参注射液OR注射用血栓通OR田七人参注射剂OR 圣泰 |
| Xuesaitong injection OR Xuesaitong zhusheye | 血塞通注射液OR血塞通注射剂OR注射用血塞通OR络泰 |
| Shuxuening injection OR Shuxuening zhusheye OR Ginkgo biloba injection OR Ginkgo leaf injection | 雪畅OR雪乐通OR舒血宁OR舒血宁注射液OR舒血宁注射剂OR注射用舒血宁OR银杏叶注射液 |
| Dengzhanxixin Injection OR Dengzhanxixin zhusheye OR Erigeron breviscapus injection | 灯盏细辛OR灯盏细辛注射液OR灯盏细辛注射剂OR灯盏细辛冻干粉 |
| Breviscapine injection OR Dengzhanhuasu Injection OR Dengzhanhuasu zhusheye | 灯盏花素OR灯盏花素注射液OR灯盏花素注射剂OR注射用灯盏花素OR龙津 |
| Huangqi Injection OR Huangqi zhusheye OR Astragalus Injection OR Radix Astragali injection | 黄芪注射液OR黄芪注射剂OR注射用黄芪冻干粉 |
| Shuxuetong injection OR Shuxuetong zhusheye | 疏血通OR疏血通注射剂OR疏血通注射液OR注射用疏血通 |
| Danhong injection OR Danhong zhusheye | 倍通OR丹红注射液OR丹红注射剂OR注射用丹红 |
| Ginkgo dipyridolum injection OR Yinxingdamo injection OR Yinxingdamo zhusheye | 银杏达莫注射液OR银杏达莫注射剂OR银杏达莫OR注射用银杏达莫OR 杏丁注射液 OR杏丁注射剂OR 美怡欣 |
| Tanshinone type IIA sulfonate injection | 丹参酮OR诺新康OR丹参酮IIA磺酸钠注射液OR丹参酮IIA磺酸钠注射剂OR注射用丹参酮IIA磺酸钠 |
| Mailuoning injection OR Mailuoning zhusheye | 脉络宁OR脉络宁注射液OR脉络宁注射剂OR注射用脉络宁 |
| Diemailing Injection OR Die-mailing injection OR Die-mailing zhusheye OR Kudiezi Injection OR Ku diezi zhusheye | 碟脉灵OR苦碟子OR碟脉灵注射液OR碟脉灵注射剂OR苦碟子注射液OR苦碟子注射剂OR悦安欣 |
| Shenmai injection OR Shenmai zhusheye | 参麦注射液OR参麦注射剂OR注射用参麦 |
| Salvianolate injection | 丹参多酚酸OR丹参多酚酸注射液OR丹参多酚酸注射剂OR丹参多酚酸盐注射液OR丹参多酚酸盐注射剂OR注射用丹参多酚酸 OR 注射用丹参多酚酸盐 |
| Safflower yellow injection | 红花黄色素OR红花黄色素注射液OR红花黄色素注射剂OR注射用红花黄色素OR上善OR 乐坦 |
| Shenxiong glucose injection OR Shenxiong injection | 佰塞通OR参芎注射液OR参芎注射剂OR参芎葡萄糖注射液 OR 参芎葡萄糖注射剂 |
| Danshenchuanxiongqin Injection OR Salvia ligustrazin injection | 恤彤OR丹参川芎嗪注射液OR丹参川芎嗪注射剂OR注射用丹参川芎嗪OR血通 |
| Guhong injection OR Guhong zhusheye | 金特佳OR谷红注射液OR谷红注射剂 |
| Gegensu Zhusheye OR Puerarin Injection OR Gegensu injection | 麦普宁OR安欣达OR葛根素注射液OR葛根素注射剂OR注射用葛根素 |
| Ciwujia injection OR Acanthopanax Senticosus injection | 刺五加注射液OR刺五加注射剂 |
| Danshen injection OR Salvia miltiorrhiza injection | 丹参注射液OR丹参注射剂OR注射用丹参 |
| Extract of ginkgo biloba injection OR Jinnaduo zhusheye OR Jinnaduo injection OR Ginkgo biloba injection OR Ginkgo biloba zhusheye | 银杏叶提取物注射液OR银杏叶提取物注射剂OR银杏注射液OR银杏注射剂OR金纳多OR注射用银杏叶提取物 |
| Perhexiline injection OR Guanxinning Injection OR Guanxinning zhusheye | 冠心宁注射液 OR 冠心宁注射剂 OR 冠心宁 |
| Qingkailing Injection OR Qingkailing zhusheye | 清开灵注射液 OR 清开灵 OR 清开灵注射剂 OR 注射用清开灵 |
| Hongjingtian Zhusheye OR Hongjingtian injection | 红景天注射液，红景天注射剂，通化玉圣，注射用红景天 |
| Shenqifuzheng injection OR Shenqifuzheng zhusheye | 参芪扶正注射液，参芪扶正注射剂，注射用参芪扶正，参芪扶正 |
| Xuebijing injection OR Xuebijing zhusheye | 血必净，血必净注射液，血必净注射剂 |
| Lulutong injection OR Lulutong zhusheye | 路路通，路路通注射液，路路通注射剂 |
| Hirudin injection OR Hirudin zhusheye OR Shuizhi Injection OR Leech injection | 水蛭注射液，水蛭注射剂 |
| Musk injection OR Shexiang injection OR Shexiang zhusheye | 麝香注射液，麝香注射剂 |
| Earthworm injection OR Earthworm zhusheye | 地龙注射液，地龙注射剂 |
| Gualoupi injection OR Gualoupi zhusheye | 瓜蒌皮，瓜蒌皮注射液，瓜蒌皮注射剂 |
| Danxiang Guanxin injection OR Danxiang Guanxin zhusheye | 丹香冠心，丹香冠心注射液，丹香冠心注射剂 |
| Xingnaojing injection OR Xingnaojing zhusheye | 醒脑静，醒脑静注射液，醒脑静注射剂 |
