# Supplementary material for: Comparative efficacy of Chinese herbal injections for treating acute cerebral infarction: a network meta-analysis of randomized controlled trials
Source: BMC Complement Altern Med. 2018 Apr 3;18:120. doi: 10.1186/s12906-018-2178-9 (PMC5883592; doi:10.1186/s12906-018-2178-9)
Supplement: Supplementary file 2 — Search strategy. (DOC 18 kb) [file 12906_2018_2178_MOESM2_ESM.doc]

Search Strategy

#1 "Brain Infarction"[Mesh]

#2 "cerebral infarction"[Title/Abstract]) OR "stoke"[Title/Abstract]) OR "brain embolism"[Title/Abstract]) OR "Ischemic stroke"[Title/Abstract]) OR "cerebrovascular disorders"[Title/Abstract]

#3 #1 OR #2

#4 "Compound danshen injection"[Title/Abstract]) OR "Composite salvia miltiorrhiza injection"[Title/Abstract]) OR "Fufang Danshen injection"[Title/Abstract]) OR "Fufang Danshen zhusheye"[Title/Abstract]

#5 "Safflor injection"[Title/Abstract]) OR "Honghua injection"[Title/Abstract]) OR "Honghua zhusheye"[Title/Abstract]

#6 "Tanshinone type IIA sulfonate injection"[Title/Abstract]

#7 "Mailuoning injection"[Title/Abstract]) OR "Mailuoning zhusheye"[Title/Abstract]

#8 "Die Mai Ling Injection"[Title/Abstract]) OR "Die-mailing injection"[Title/Abstract]) OR "Die-mailing zhusheye"[Title/Abstract]) OR "Ku die zi Injection"[Title/Abstract]) OR "Ku die zi zhusheye"[Title/Abstract]

#9 "Shenmai injection"[Title/Abstract]) OR "Shenmai zhusheye"[Title/Abstract]

#10 "Salvianolate injection"[Title/Abstract]

#11 "Safflower yellow injection"[Title/Abstract]

#12 "Shenxiong glucose injection"[Title/Abstract]) OR "Shenxiong injection"[Title/Abstract]

#13 "Danshenchuanxiongqin Injection"[Title/Abstract] OR "Salvia ligustrazin injection "[Title/Abstract]

#14 "Guhong injection"[Title/Abstract]) OR "Guhong zhusheye"[Title/Abstract]

#15 "Gegensu Zhusheye"[Title/Abstract]) OR "Puerarin Injection"[Title/Abstract]) OR "Gegensu injection"[Title/Abstract]

#16 "Ciwujia injection"[Title/Abstract]) OR "Acanthopanax Senticosus injection"[Title/Abstract]

#17 "Danshen injection"[Title/Abstract]) OR "Salvia miltiorrhiza injection"[Title/Abstract]

#18 "Extract of ginkgo biloba injection"[Title/Abstract]) OR "Jinnaduo zhusheye"[Title/Abstract]) OR "Jinnaduo injection"[Title/Abstract]) OR "Ginkgo biloba injection"[Title/Abstract]) OR "Ginkgo biloba zhusheye"[Title/Abstract]

#19 "Perhexiline injection"[Title/Abstract]) OR "Guanxinning Injection"[Title/Abstract]) OR "Guanxinning zhusheye"[Title/Abstract]

#20 "Qingkailing Injection"[Title/Abstract]) OR "Qingkailing zhusheye"[Title/Abstract]

#21 "Hongjingtian Zhusheye"[Title/Abstract]) OR "Hongjingtian injection"[Title/Abstract]

#22 "Shenqifuzheng injection"[Title/Abstract]) OR "Shenqifuzheng zhusheye"[Title/Abstract]

#23 "Xuebijing injection"[Title/Abstract]) OR "Xuebijing zhusheye"[Title/Abstract]

#24 "Lulutong injection"[Title/Abstract]) OR "Lulutong zhusheye"[Title/Abstract]

#25 "hirudin injection"[Title/Abstract]) OR "hirudin zhusheye"[Title/Abstract]) OR "Shuizhi Injection"[Title/Abstract]) OR "leech injection"[Title/Abstract]

#26 "Musk injection"[Title/Abstract]) OR "Shexiang injection"[Title/Abstract]) OR "Shexiang zhusheye"[Title/Abstract]

#27 "Earthworm Injection"[Title/Abstract]) OR "Earthworm zhusheye"[Title/Abstract]

#28 "Gualoupi injection"[Title/Abstract]) OR "Gualoupi zhusheye"[Title/Abstract]

#29"Danxiang Guanxin injection"[Title/Abstract]) OR "Danxiang Guanxin zhusheye"[Title/Abstract]

#30 "Xingnaojing injection"[Title/Abstract]) OR "Xingnaojing zhusheye"[Title/Abstract]

#31 "Danhong injection"[Title/Abstract]) OR "Danhong zhusheye"[Title/Abstract]

#32 "Shuxuetong injection"[Title/Abstract]) OR "Shuxuetong zhusheye"[Title/Abstract]

#33 "Huangqi Injection"[Title/Abstract]) OR "Huangqi zhusheye"[Title/Abstract]) OR "Astragalus Injection"[Title/Abstract]) OR "Radix Astragali injection"[Title/Abstract]

#34 "Breviscapine injection"[Title/Abstract]) OR "Dengzhanhuasu Injection"[Title/Abstract]) OR "Dengzhanhuasu zhusheye"[Title/Abstract]

#35 "Dengzhanxixin Injection"[Title/Abstract]) OR "Dengzhanxixin zhusheye"[Title/Abstract]) OR "Erigeron breviscapus injection"[Title/Abstract]

#36 "Shuxuening injection"[Title/Abstract]) OR "Shuxuening zhusheye"[Title/Abstract]) OR "ginkgo biloba injection"[Title/Abstract]) OR "ginkgo leaf injection"[Title/Abstract]

#37 "Sanqi Panax Notoginseng"[Title/Abstract]) OR "Xueshuantong Injection"[Title/Abstract]) OR "Xueshuantong zhusheye"[Title/Abstract]) OR "Shengtaixueshuantong injection"[Title/Abstract]

#38 "Tetramethylpyrazine injection"[Title/Abstract]) OR "Chuanxiongqin"[Title/Abstract]) OR "Chuanxiongqin zhusheye"[Title/Abstract]) OR "Chuanxiongqin injection"[Title/Abstract]

#39 "Xuesaitong injection"[Title/Abstract]) OR "Xuesaitong zhusheye"[Title/Abstract]

#40 "Ginkgo dipyridolum injection"[Title/Abstract]) OR "Yinxingdamo injection"[Title/Abstract]) OR "Yinxingdamo zhusheye"[Title/Abstract]

#41 #4 OR #5 OR #6 OR #7 OR #8 OR #9 OR #10 OR #11 OR #12 OR #13 OR #14 OR #15 OR #16 OR #17 OR #18 OR #19 OR #20 OR #21 OR #22 OR #23 OR #24 OR #25 OR #26 OR #27 OR #28 OR #29 OR #30 OR #31 OR #32 OR #33 OR #34 OR #35 OR #36 OR #37 OR #38 OR #39 OR #40

#42 randomized controlled trial[Publication Type]

#43 controlled clinical trial[Publication Type]

#44 randomized[Title/Abstract]

#45 placebo[Title/Abstract]

#46 randomly[Title/Abstract]

#47 trial[Title/Abstract]

#48 groups[Title/Abstract]

#49 drug therapy[MeSH Terms]

#50 "drug therapy" [Subheading]

#51 #43 OR #44 OR #45 OR #46 OR #47 OR #48 OR #49 OR #50

#52 animals [MeSH Terms]

#53 humans[MeSH Terms]

#54 #52 NOT #53

#55 #51 NOT #54

#56 #3 AND #41 AND #55
