# Supplementary material for: Comparative efficacy of Chinese herbal injections for treating acute cerebral infarction: a network meta-analysis of randomized controlled trials
Source: BMC Complement Altern Med. 2018 Apr 3;18:120. doi: 10.1186/s12906-018-2178-9 (PMC5883592; doi:10.1186/s12906-018-2178-9)
Supplement: Supplementary file 3 — More details about the product information of CHIs. (DOC 42 kb) [file 12906_2018_2178_MOESM3_ESM.doc]

**Supplementary file 2. More details about the product information of CHIs**

| CHI name | Raw materials | Labeled efficacy | Indications |
| --- | --- | --- | --- |
| Shuxuening injection | *Ginkgo Folium* | Dilating blood vessels, improving microcirculation | Ischemic cardiac-cerebral vascular diseases, coronary disease, stenocardia, cerebral embolism, cerebral vasospasm, etc. |
| Shuxuetong injection | *Hirudo, Pheretima* | Promoting blood circulation for removing obstruction in collaterals | Ischemic stroke at acute stage with hemiplegia and facial paralysis, acute cerebral infarction. |
| Shenxiong injection | Ligustrazine Hydrochloride*, Salviae Miltiorrhizae Radix et Rhizoma* | Promoting blood circulation for removing blood stasis, removing obstruction in collaterals for nourishing heart | Cerebrovascular disease obliteration and other ischemic vascular diseases. |
| Mailuoning injection | *Lonicerae Japonicae Flos, Achyranthis Bidentatae Radix, Dendrobii Caulis, Scrophulariae Radix* | Clearing heat for nourishing yin, promoting blood circulation for removing blood stasis | Thromboangitis obliterans, arteriosclerosis obliteration, phlebothrombosis, cerebral thrombosis and its sequelae. |
| Honghuahuangsesu injection | Carthamin yellow | Promoting blood circulation, removing blood stasis, removing obstruction in collaterals | Coronary heart disease with stable angina. |
| Fufangdanshen injection | *Salviae Miltiorrhizae Radix et Rhizoma, Dalbergiae Odoriferae Lignum* | Promoting blood circulation for removing blood stasis, regulating qi-flowing for relieving pain | Angina, myocardial infarction, cerebral anoxia, cerebral embolism, neurasthenia, etc. |
| Dengzhanhuasu injection | Breviscapine | Promoting blood circulation for removing blood stasis, removing obstruction in collaterals for relieving pain | Stroke and its sequelae, angina, coronary heart disease. |
| Dengzhanxixin injection | *Erigerontis Herba* | Promoting blood circulation for removing blood stasis, removing obstruction in collaterals for relieving pain | Obstruction by blood stasis, apoplectic hemiplegia, facial paralysis, dysphasia, chest stuffiness and pains; ischemic stroke and coronary heart disease with angina pectoris with above symptoms. |
| Danshenchuanxiongqin injection | Tanshinol, ligustrazine | Promoting blood circulation for removing blood stasis | Occlusive cerebrovascular diseases, such as cerebral circulation insufficiency, cerebral thrombosis, cerebral embolism and other ischemic cardiovascular diseases, such as chest distress, angina pectoris, myocardial infarction, ischemic stroke, thrombotic obliterans and so on. |
| Danshen injection | *Salviae Miltiorrhizae Radix et Rhizoma* | Promoting blood circulation for removing blood stasis, removing obstruction in collaterals for nourishing heart | Coronary heart disease with chest tightness, angina pectoris. |
| Danhong injection | *Salviae Miltiorrhizae Radix et Rhizoma, Carthami Flos* | Promoting blood circulation for removing obstruction in collaterals | Chest pain and stroke caused by blood stasis obstruction and including chest pain, chest tightness, heart palpitations, facial paralysis, dysphasia, activity inconvenience and other symptoms; coronary heart disease, angina pectoris, myocardial infarction, blood stasis type pulmonary heart disease, ischemic encephalopathy, cerebral thrombosis |
| Yinxingdamo injection | Total flavone of Ginkgo, dipyridamole | Dilating coronary vessels and cerebral vessels, inhibiting the aggregation of platelets | To prevent and treat coronary heart disease and thromboembolic disease. |
| Ligustrazine injection | Ligustrazine hydrochloride | Inhibiting the aggregation of platelets, dilating blood vessels, improving microcirculation | Occluded cerebrovascular diseases such as cerebral circulation insufficiency, cerebral thrombosis, cerebral embolism and other ischemic vascular diseases such as coronary heart disease, vasculitis, etc. |
| Xuesaitong injection | Panax notoginseng saponins | Promoting blood circulation for removing obstruction in collaterals | Central retinal vein occlusion, hyphema, glaucoma, sequelae of cerebrovascular disease, viral hepatitis. |
| Xueshuantong injection | Panax notoginseng saponins | Promoting blood circulation for removing blood stasis, dilating blood vessels, improving microcirculation | Central retinal vein occlusion, hyphema, sequelae of cerebrovascular disease, internal ophthalmopathy. |
