# Supplementary figures and images for: Comparative efficacy of Chinese herbal injections for treating acute cerebral infarction: a network meta-analysis of randomized controlled trials
Source: BMC Complement Altern Med. 2018 Apr 3;18:120. doi: 10.1186/s12906-018-2178-9 (PMC5883592; doi:10.1186/s12906-018-2178-9)

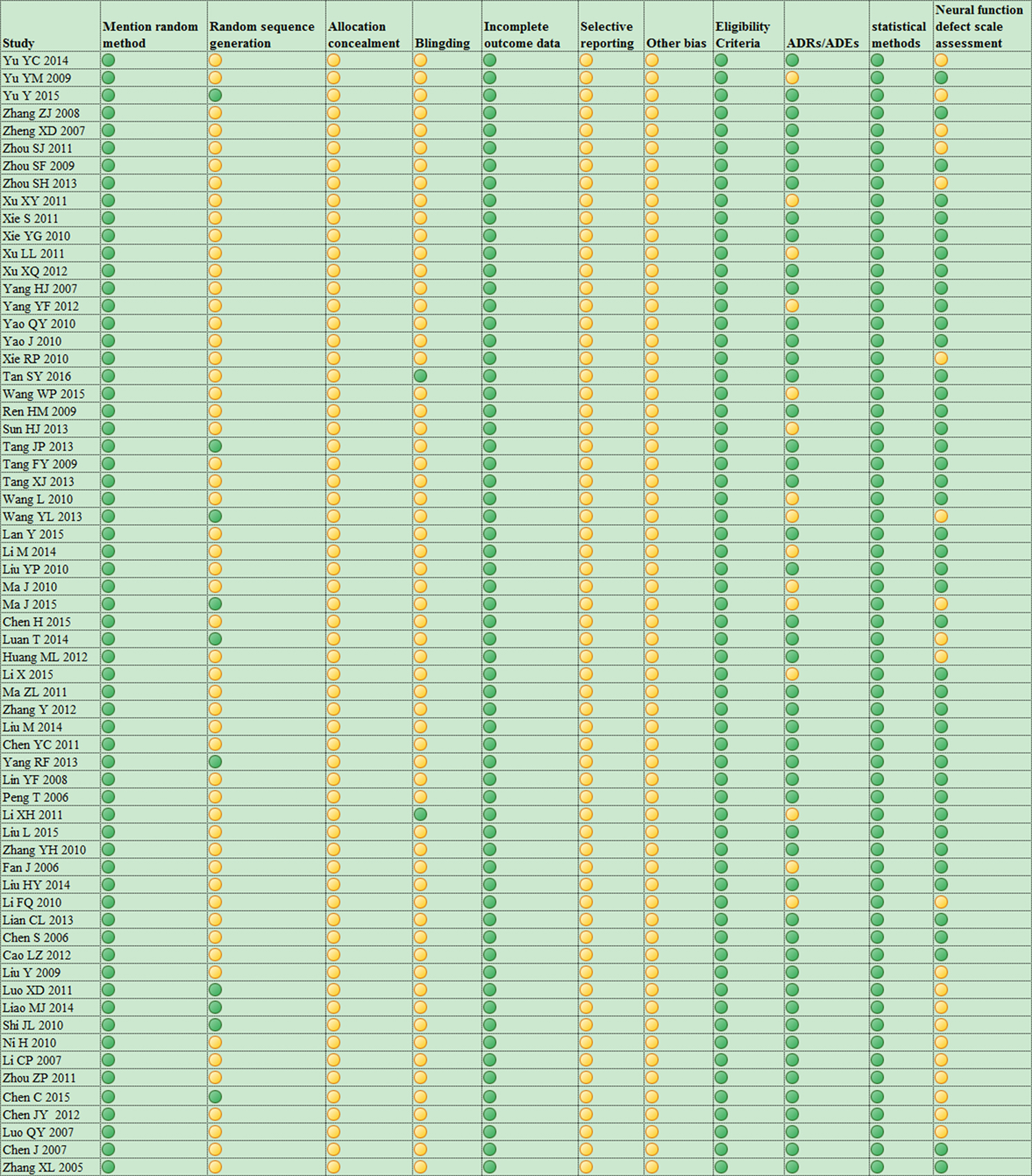

Supplement: Supplementary file 4 — Figure S1. Risk of bias summary. Note: Green: low risk of bias; Yellow: unclear risk of bias; Red: high risk of bias. (JPEG 1934 kb) [file 12906_2018_2178_MOESM4_ESM.jpg]

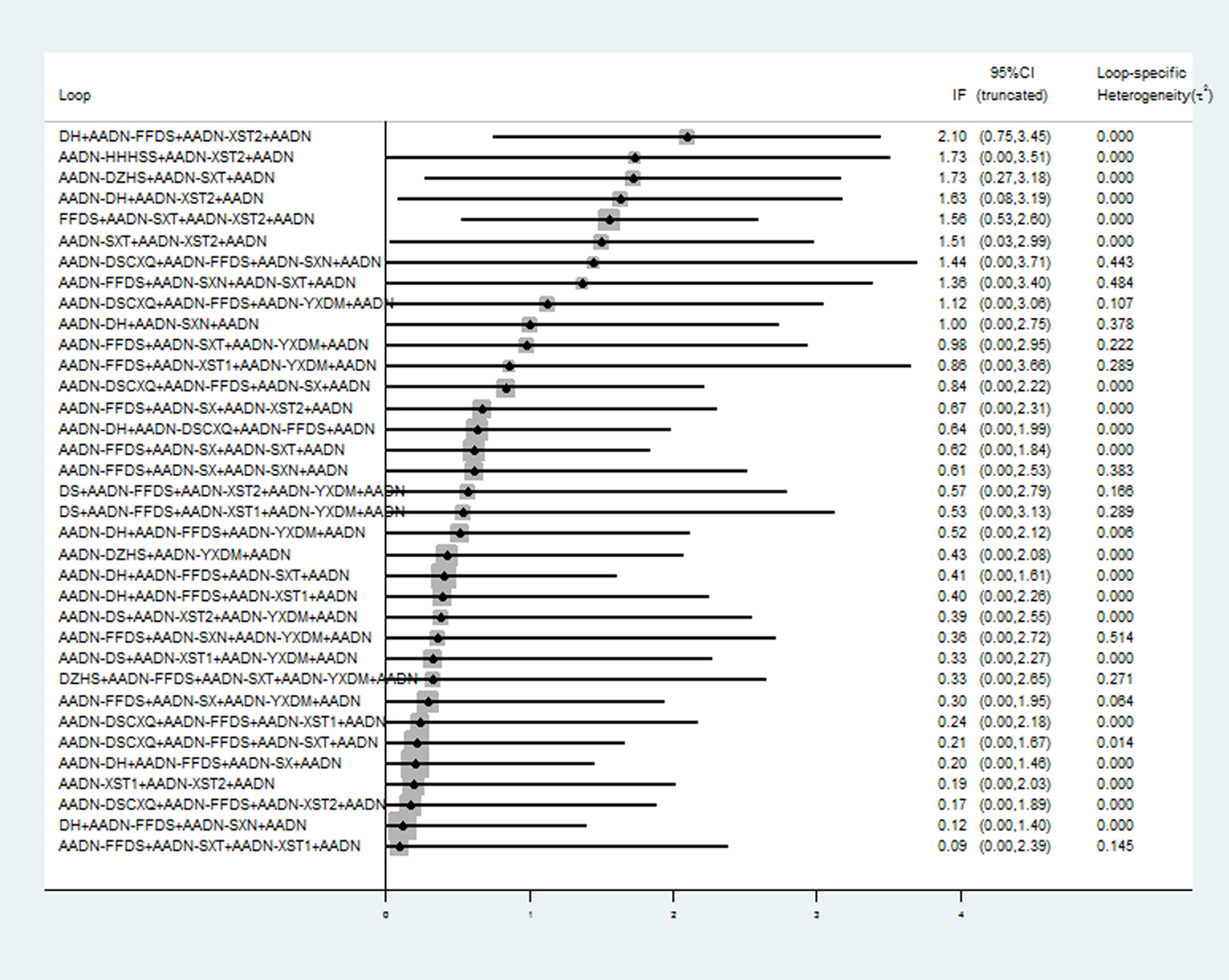

Supplement: Supplementary file 5 — Figure S2. Inconsistency test for the markedly effective rate. (JPEG 953 kb) [file 12906_2018_2178_MOESM5_ESM.jpg]

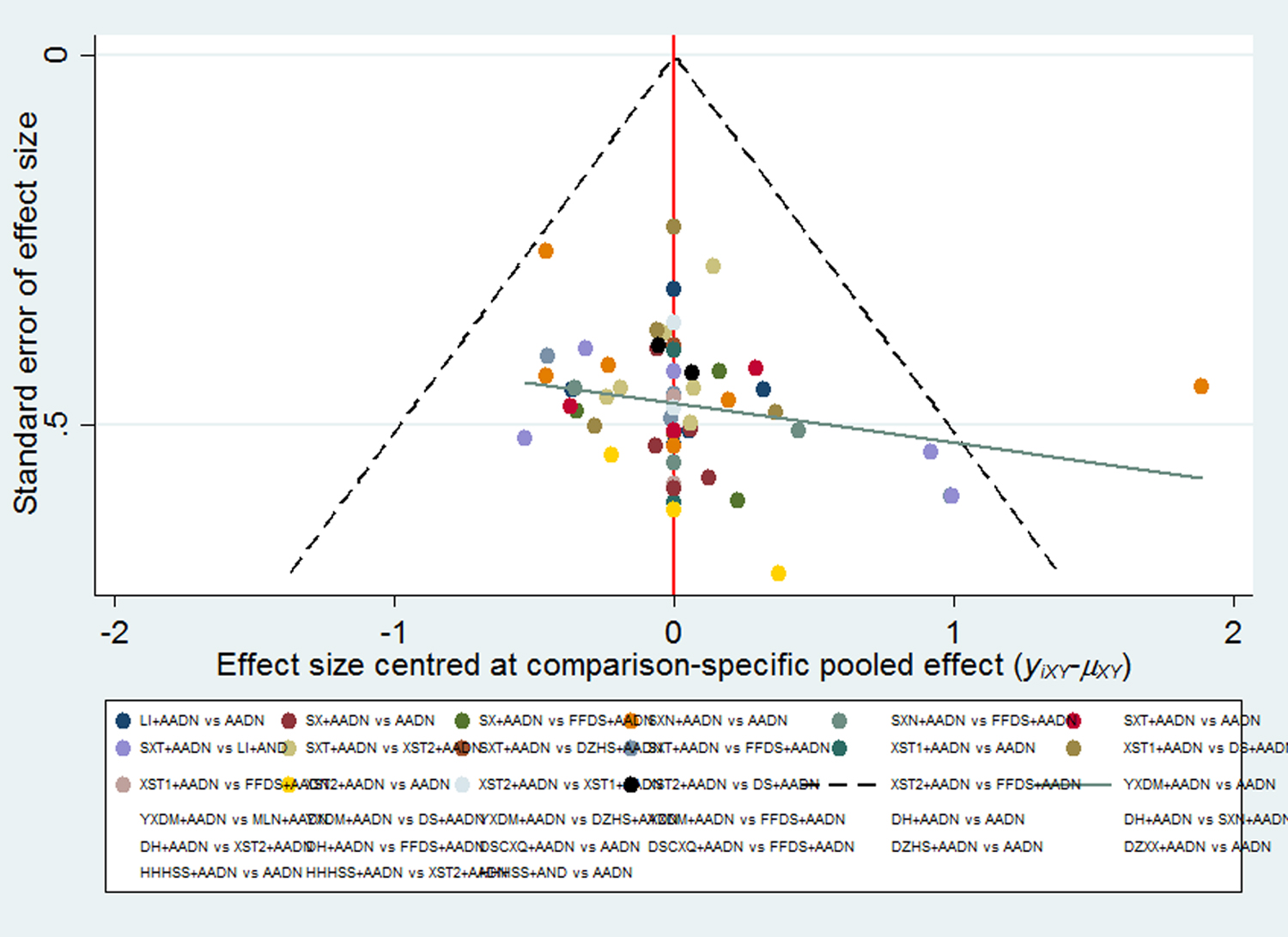

Supplement: Supplementary file 6 — Figure S3. Comparison adjusted funnel plot. (JPEG 533 kb) [file 12906_2018_2178_MOESM6_ESM.jpg]

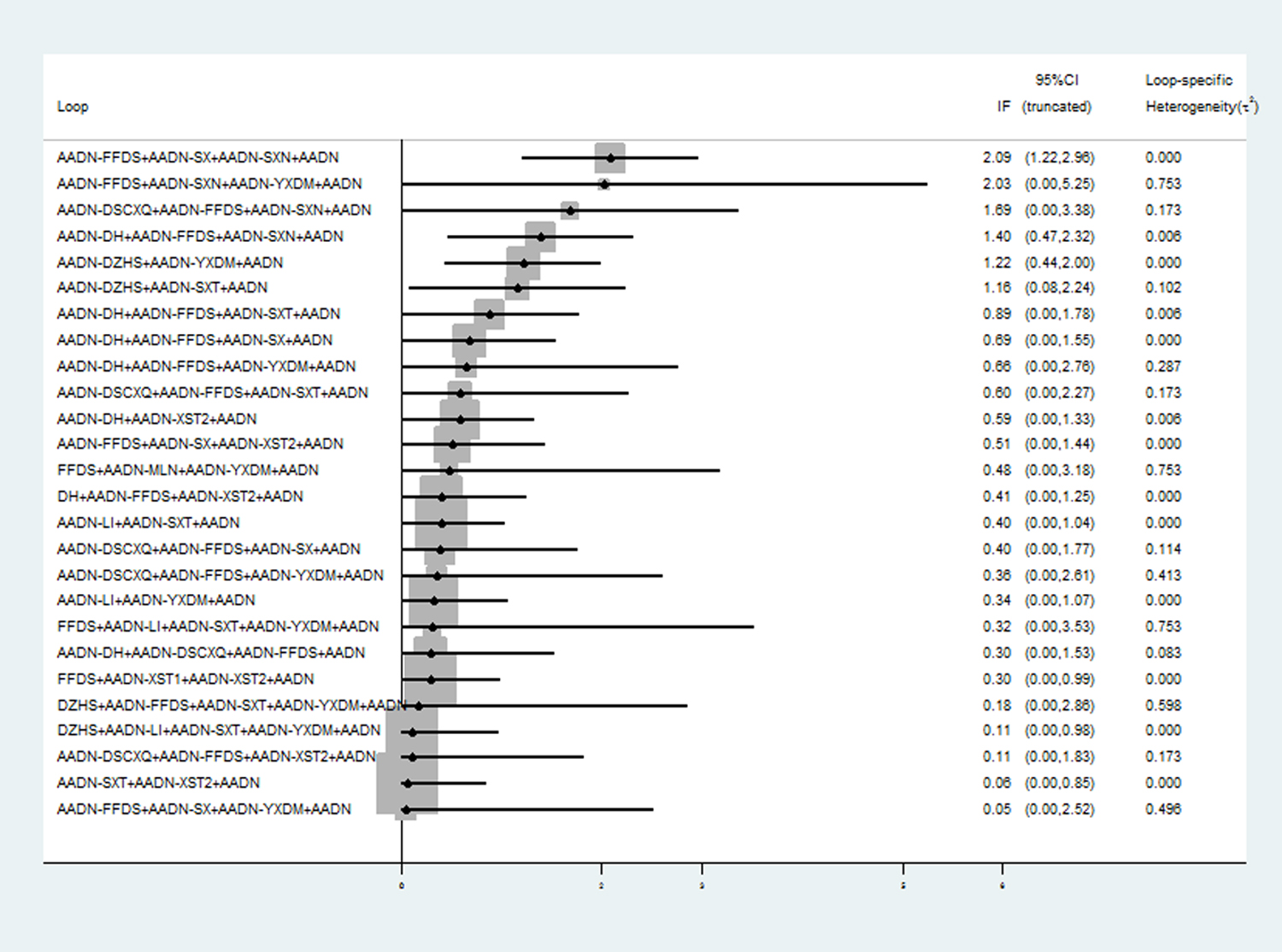

Supplement: Supplementary file 7 — Figure S4. Inconsistency test for improvement of neurological impairment. (JPEG 693 kb) [file 12906_2018_2178_MOESM7_ESM.jpg]
